# Enhancing the ROS Sensitivity of a Responsive Supramolecular Hydrogel Using Peroxizyme Catalysis

**DOI:** 10.1021/acs.biomac.3c00262

**Published:** 2023-06-23

**Authors:** Irene Piergentili, Thomas Hilberath, Benjamin Klemm, Frank Hollmann, Rienk Eelkema

**Affiliations:** † Department of Chemical Engineering, Delft University of Technology, Van der Maasweg 9, 2629 HZ Delft, The Netherlands; ‡ Department of Biotechnology, Delft University of Technology, Van der Maasweg 9, 2629 HZ Delft, The Netherlands

## Abstract

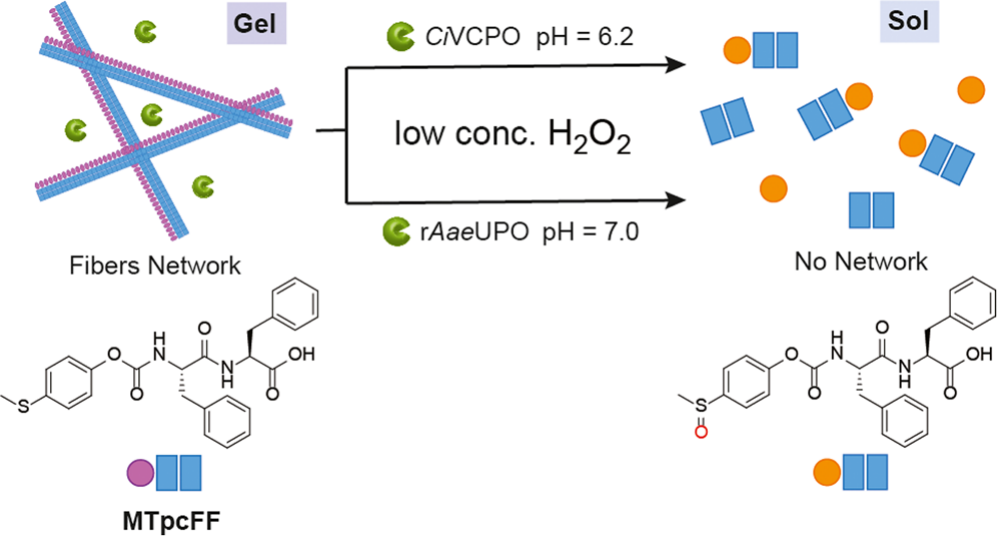

Hydrogels that can disintegrate upon exposure to reactive oxygen species (ROS) have the potential for targeted drug delivery to tumor cells. In this study, we developed a diphenylalanine (FF) derivative with a thioether phenyl moiety attached to the N-terminus that can form supramolecular hydrogels at neutral and mildly acidic pH. The thioether can be oxidized by ROS to the corresponding sulfoxide, which makes the gelator hydrolytically labile. The resulting oxidation and hydrolysis products alter the polarity of the gelator, leading to disassembly of the gel fibers. To enhance ROS sensitivity, we incorporated peroxizymes in the gels, namely, chloroperoxidase *Ci*VCPO and the unspecific peroxygenase r*Aae*UPO. Both enzymes accelerated the oxidation process, enabling the hydrogels to collapse with 10 times lower H_2_O_2_ concentrations than those required for enzyme-free hydrogel collapse. These ROS-responsive hydrogels could pave the way toward optimized platforms for targeted drug delivery in the tumor microenvironment.

## Introduction

Hydrogels made by self-assembly of small molecules have been drawing attention for over two decades due to the perspective of using these soft materials for applications ranging from drug delivery to tissue engineering.^1,2^ The initial fascination over serendipitously discovered gels was soon replaced by the need to understand the forces that drive gel formation in a way to design a priori molecules that are able to gelate.^3,4^ Considering the pool of self-assembled structures in biological systems,^5^ nature was once again where researchers found inspiration. The first example of peptide-based low-molecular-weight gelator (LMWG), reported in 1995, was used to realize a thermoreversible gel as a carrier for antigen delivery and presentation.^6^ Then, the recognition of diphenylalanine (**FF**) as a basic structure able to form ordered nanostructures^7^ led to the derivatization of this sequence to obtain a large variety of novel hydrogelators.^8−11^ The biocompatibility, the possibility of bottom-up fabrication, and easy chemical modification made diphenylalanine the natural choice as a short peptide sequence for biomedical materials.^12−14^ Subsequently, this versatile building block was functionalized with groups sensitive to various stimuli such as pH,^15,16^ enzymes,^17,18^ ultraviolet (UV) light,^19,20^ and redox change^21^ to realize responsive hydrogels. Reactive oxygen species (ROS) overproduction is typical in many tumor and diseased cells, causing a redox imbalance in the microenvironment. Ikeda and co-workers prepared **FF**-type peptides with a boronoaryl group, which fragments in the presence of hydrogen peroxide (H_2_O_2_), one of the most used ROS.^22^

**Figure 1:**
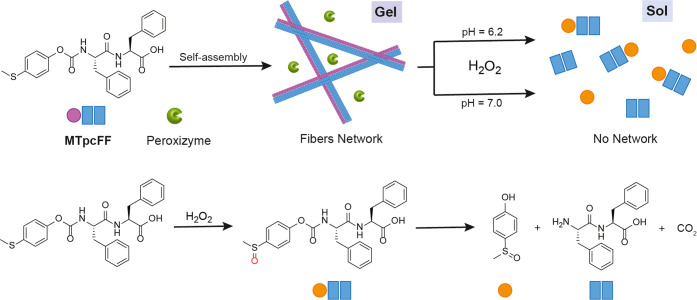
Schematic representation of the **MTpcFF** self-assembly with peroxizymes showing the formation of a nanofiber network (gel) and H_2_O_2_-triggered gel–sol transition. Peroxizymes: gels with *Ci*VCPO are prepared in citrate buffer at pH = 6.2, and gels with r*Aae*UPO are prepared in phosphate buffer at pH = 7.0.

Besides the boronates, sulfides are commonly used oxidation-sensitive groups for stimuli-triggered nanomedicine.^23,24^ In the aqueous medium, the oxidation of a sulfide into sulfoxide or sulfone produces a switch in solubility that can be used to destabilize self-assembled structures bearing these moieties.^25^ The solubility switch from thioether to sulfoxide to trigger the gel–sol transition of hydrogels is not unprecedent,^26−28^ but to the best of our knowledge, it has not been used for short peptide-based gelators.^29,30^ Recently, we developed a thioether phenyl ester-based cascade mechanism in which the oxidation to sulfoxide induces the hydrolysis of the ester.^31^ We functionalized diphenylalanine with the thioether phenyl moiety, aiming to realize a hydrogelator able to respond to H_2_O_2_ through a logic gate mechanism that enables the gel to disrupt. Thus, we synthesized **MTpcFF** (Figure 1), an aromatic amphiphile able to form a self-assembled network in an aqueous environment at both pH values 6.2 and 7.0, which are favorable conditions for biological applications. Moreover, once demonstrated the responsiveness toward H_2_O_2_, we included two different H_2_O_2_-dependent enzymes, namely, the vanadium-dependent chloroperoxidase from *Curvularia inaequalis* (*Ci*VCPO) and the unspecific peroxygenase from *Agrocybe aegerita* (r*Aae*UPO, PaDaI mutant) in the hydrogel. Chloroperoxidases are enzymes that catalyze the formation of hypohalites from H_2_O_2_ and halides. Despite their lower oxidation potential compared to H_2_O_2_ (1.78 V), hypohalites such as HOCl (1.49 V) and HOBr (1.34 V) are known to increase the rate of sulfide oxidation.^32−34^ Among the chloroperoxidases used for oxidation of thioanisole-type moieties, we chose *Ci*VCPO for its robustness and high catalytic activity even in the presence of an organic solvent.^35−38^ The production of HOCl is optimal at pH below 7;^39^ therefore, this system is suitable for mildly acidic conditions, which are often present in tumor tissues.^40^ In the **MTpcFF** gel, we also incorporated r*Aae*UPO, which can catalyze the oxidation of aromatic sulfides with H_2_O_2_ at neutral pH^41,42^ and it is known to have an enzymatic activity in water with a cosolvent and even in a pure organic solvent.^43,44^

Despite the different mechanisms of the two peroxizymes, we found that their influence over the gel–sol transition of **MTpcFF** hydrogels upon the addition of H_2_O_2_ is comparable. This establishes a versatile strategy that allows us to obtain ROS-responsive hydrogels in both mildly acidic and neutral conditions.

## Experimental Section

### Materials

Diphenylalanine (FF) and triphenyl alanine (FFF) were purchased from Sigma-Aldrich. 4-(Methylthio)phenol and 4-(methylsulfonyl)phenol were purchased from Sigma-Aldrich and TCI, respectively.

### Preparation of *Ci*VCPO

The vanadium-dependent chloroperoxidase from *C. inaequalisCi*VCPO was produced in recombinant *Escherichia coli* TOP10 pBADgIIIB VCPO following previously described procedures.^45^*Ci*VCPO was purified via heat treatment according to a recently reported protocol.^46^

### Preparation of r*Aae*UPO

The expression-engineered variant of the recombinant unspecific peroxygenase from *A. aegerita* (r*Aae*UPO, PaDaI mutant) was used as a concentrated supernatant derived from a 2500 L pilot-scale cultivation of recombinant *Pichia pastoris* X-33.^47,48^

### Synthesis of 4-(Methylthio)phenyl 4-Nitrophenyl Carbonate

4-Nitrophenyl chloroformate (0.97 g, 4.80 mmol) and 4-(methylthio)phenol (0.56 g, 4.00 mmol) were dissolved in 30 mL of dichloromethane, and the solution was cooled to 0 °C in an ice bath. Triethylamine (0.67 mL, 4.80 mmol) was added dropwise; the mixture was allowed to warm to room temperature and stirred for 4 h. TLC analysis (silica; eluant: dichloromethane) indicated that no 4-(methylthio)phenol remained. The reaction mixture was washed with water (2 × 25 mL) and brine (25 mL). The organic layer was dried over Na_2_SO_4_, filtered, and the solvent was removed by rotatory evaporation. Recrystallization from toluene (160 mL) gave the pure product (0.92 g, yield 77%) as a yellow solid. ^1^H NMR (400 MHz, CDCl_3_) δ = 8.32 (d, *J* = 9.2 Hz, 2H), 7.48 (d, *J* = 9.2 Hz, 2H), 7.31 (d, *J* = 8.8 Hz, 2H), 7.21 (d, *J* = 8.8 Hz, 2H), 2.50 (s, 3H). ^13^C NMR (101 MHz, CDCl_3_) δ = 155.39, 151.18, 148.42, 145.78, 137.24, 127.98, 125.56, 121.86, 121.32, 16.38. Spectroscopic data aligned with those reported in the literature.^49^

### Synthesis of 2-(((4-(Methylthio)phenoxy)carbonyl)amino)ethane-1-sulfonic Acid (**1**)

To a solution of 4-(methylthio)phenyl 4-nitrophenyl carbonate (0.15 g, 0.50 mmol) in THF (2.0 mL) was added dropwise taurine (0.09 g, 0.75 mmol) and DIPEA (0.13 mL, 0.75 mmol) in distilled water (2.0 mL) at 0 °C. The reaction mixture was allowed to warm at room temperature and stirred overnight.^50,51^ After the removal of THF by rotatory evaporation, the mixture was extracted with ethyl acetate to remove the excess of *p*-nitrophenol. The aqueous layer was freeze-dried overnight. The crude was dissolved in acetonitrile, allowing the precipitation of the free taurine as a white solid. After filtration through syringe filters (45 μm), the acetonitrile was evaporated in vacuo. The mixture was dissolved in BuOH and further purified by flash chromatography over silica gel (CH_3_COOH/H_2_O/BuOH 5:5:90) to remove the excess of DIPEA. Compound **1** was obtained (0.07 g, yield 48%) as a slightly yellow solid. ^1^H NMR (400 MHz, CD_3_OD) δ = 7.29 (d, *J* = 8.6 Hz, 2H), 7.07 (d, *J* = 8.6 Hz, 2H), 3.62 (t, *J* = 6.9 Hz, 2H), 3.06 (t, *J* = 6.9 Hz, 2H), 2.48 (s, 3H). ^13^C NMR (101 MHz, CD_3_OD) δ = 156.8, 150.4, 136.6, 129.0, 123.4, 51.7, 38.3, 16.5. Liquid chromatography-mass spectrometry (LC-MS) (electrospray ionisation (ESI)) calcd for C_10_H_12_NO_5_S^–^ [M – H]^−^: 290.02, found: 290.04.

### Synthesis of MTpcFF

To a solution of 4-(methylthio)phenyl 4-nitrophenyl carbonate (0.26 g, 0.85 mmol) in THF (16.0 mL) were added dropwise FF (0.40 g, 1.28 mmol) and DIPEA (0.22 mL, 1.28 mmol) in distilled water (4.0 mL) at 0 °C. The reaction mixture was allowed to warm at room temperature and stirred overnight.^22,50^ TLC analysis confirmed that no 4-(methylthio)phenyl 4-nitrophenyl carbonate remained. After the removal of THF through rotatory evaporation, the aqueous mixture was acidified (pH = 2–3) with a 5% citric acid solution. The reaction mixture was extracted with ethyl acetate (3 × 40 mL), and the combined organic layer was washed with water. The organic layer was dried over Na_2_SO_4_ and filtered. The filtrate was concentrated and precipitated in hexane twice to provide **MTpcFF** (0.34 g, yield 84%) as a slightly yellow solid. ^1^H NMR (400 MHz, CD_3_CN) δ = 7.32–7.22 (m, 12H), 7.01 (d, *J* = 7.8 Hz, 1H), 6.89 (d, *J* = 8.5 Hz, 2H), 6.20 (d, *J* = 8.4 Hz, 1H), 4.70–4.60 (m, 1H), 4.38–4.29 (m, 1H), 3.23–3.10 (m, 2H), 3.06–2.95 (m, 1H), 2.89–2.77 (m, 1H), 2.46 (s, 3H). ^13^C NMR (101 MHz, CD_3_CN) δ = 172.7, 171.8, 155.2, 149.7, 138.2, 137.8, 130.3, 129.4, 129.3, 128.3, 127.8, 127.7, 123.2, 57.2, 54.4, 38.5, 37.8, 16.3. LC-MS (ESI) calcd for C_26_H_26_N_2_O_5_S [M + H]^+^: 479.16, found 479.13.

### MTpcFF Hydrogel Preparation

The *Ci*VCPO/**MTpcFF** gels were prepared by dissolving 0.75 mg of **MTpcFF** in 5.0 μL of DMSO in a 1.5 mL screwed vial; then 93.5 μL of citrate buffer (CB, 50 mM, pH = 6.2) and 1.5 μL of the 65.0 μM *Ci*VCPO stock solution in tris/H_2_SO_4_ buffer (50 mM, pH 8.2) were added. For enzyme-free gels, 1.5 μL of citrate buffer was added instead of the enzyme stock solution. The r*Aae*UPO/**MTpcFF** gels were prepared by dissolving 1.0 mg of **MTpcFF** in 5.0 μL of DMSO in a 1.5 mL screwed vial; then, 93.8 μL of phosphate buffer (PB, 50 mM, pH = 7.0) and 1.2 μL of the 83.9 μM r*Aae*UPO stock solution were added to the potassium phosphate buffer (20 mM, pH 7.0). For enzyme-free gels, 1.2 μL of phosphate buffer was added instead of the enzyme stock solution. Each vial was stirred by vortexing for 3 s, capped, placed on a stable surface, and left undisturbed overnight. The gelation was evaluated by turning the vial upside down.

### Rheology of MTpcFF Hydrogels

Oscillatory experiments were performed using a rheometer AR G2 from TA Instruments in a strain-controlled mode. The rheometer was equipped with a steel plate and plate geometry of diameter 25 mm and a water trap. The temperature of the plates was controlled at 25 ± 0.2 °C. The gel mixtures were prepared as reported above, obtaining a total volume for each gelation experiment of 0.1 mL. After stirring the vial by vortexing for 3 s, the gel was pipetted on the bottom plate of the rheometer and the upper plate was slowly rotated to equally spread the gel. The storage and loss moduli *G*′ and *G*″ were followed over time with the rheometer during the formation of the gel, setting up the instrument with a frequency of 1.0 Hz and under 1.0% strain. The measurements were stopped when no further increase of *G*′ was observed. A frequency sweep was measured in the range of 0.01–100 rad/s, confirming that the moduli are constant in the frequency range chosen and the strain sweep revealed that the applied strain percentage is in the linear strain regime. *G*′ was greater than *G*″, and both *G*′ and *G*″ were frequency-independent, which indicated the typical viscoelastic behavior of a hydrogel consisting of fiber networks. The rheological properties of **MTpcFF** gels were not significantly influenced by the presence of the enzymes *Ci*VCPO and r*Aae*UPO.

### H_2_O_2_ Response of MTpcFF Hydrogels with and without Peroxizymes (Tube Inversion)

To the **MTpcFF** gels prepared in CB as described above, 5.0 μL of NaCl 3.00 M was placed on top of the gels. Then, 5.0 μL of 312, 156, 78.0, or 31.0 mM H_2_O_2_ stock solution was added to provide, respectively, 1.0, 0.5, 0.25, or 0.1 equiv of H_2_O_2_ to the gels. To the **MTpcFF** gels prepared in PB as described above, 10.0 μL of 200, 100, 50.0, or 20.0 mM H_2_O_2_ stock solution was added on top of the gels to provide, respectively, 1.0, 0.5, 0.25, or 0.1 equiv of H_2_O_2_. Control experiments were performed by adding 10.0 μL of the corresponding buffer instead of H_2_O_2_ stock solution. All of the experiments were performed at 37 °C. The gel–sol transition was then visually evaluated according to the tube-inversion method over time. Photographs were acquired at different stages of the gel–sol transition. The hydrogels resulted in being stable in the absence of H_2_O_2_ over 24 h but suffered water loss due to the prolonged time at 37 °C. To maintain the concentrations of the gels constant, control experiments were stopped at 8 h.

## Results and Discussion

### Oxidation and Hydrolysis Study on a Soluble Model Compound

**Figure 2:**
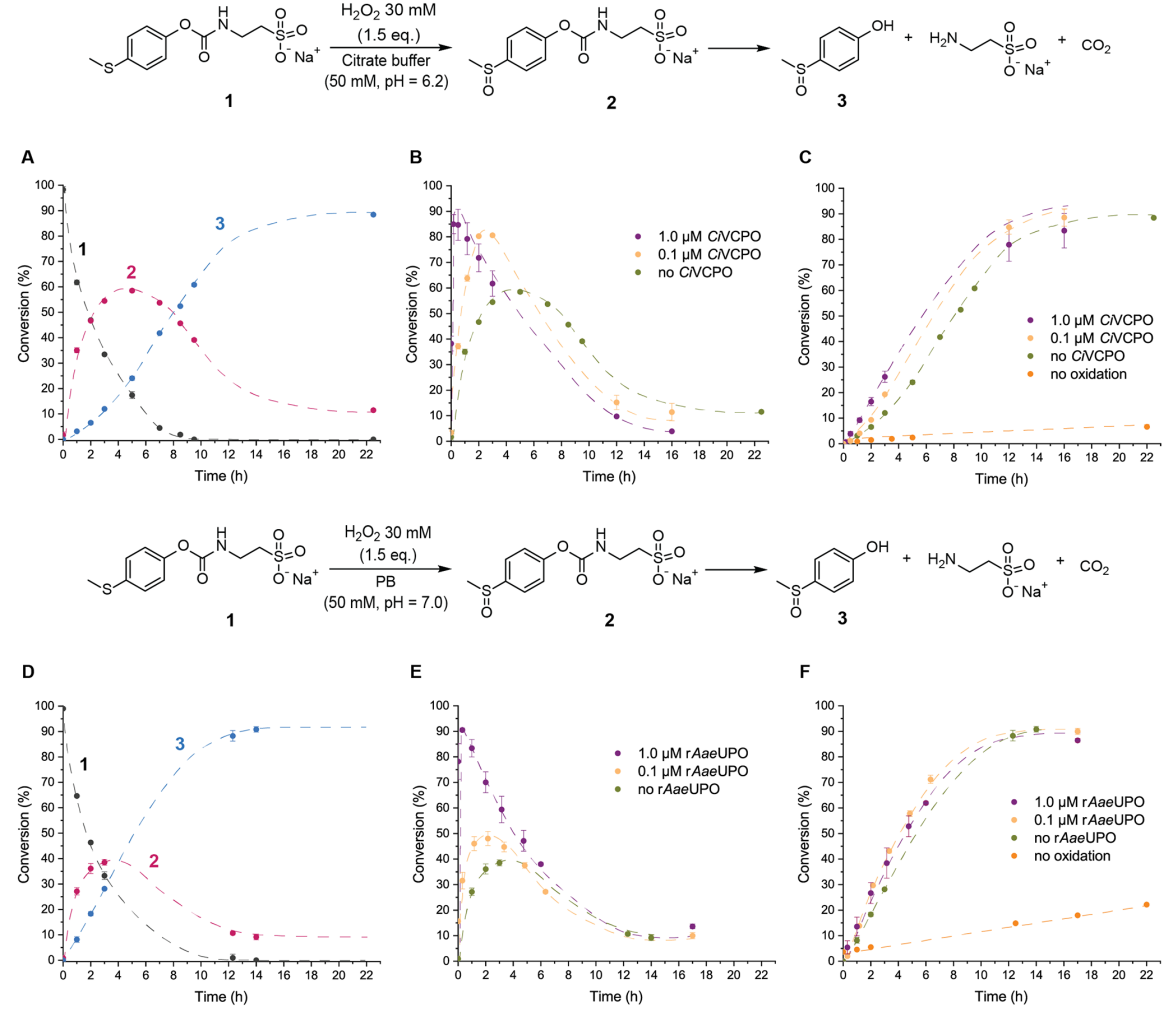
Scheme illustrating the species formed from compound **1** after the addition of H_2_O_2_ at 37 °C. (A) Conversion profile without enzyme in citrate buffer. (B) Conversion profile of **2** after the addition of H_2_O_2_ in the presence of 0.0, 0.1, and 1.0 μM *Ci*VCPO. (C) Conversion profile of **3** without H_2_O_2_ in citrate buffer (orange line, the conversion refers to 4-(methylthio)phenol) and after the addition of H_2_O_2_ in the presence of 0.0, 0.1, and 1.0 μM *Ci*VCPO. (D) Conversion profile without enzyme in phosphate buffer. (E) Conversion profile of **2** after the addition of H_2_O_2_ in the presence of 0.0, 0.1, and 1.0 μM r*Aae*UPO. (F) Conversion profile of **3** in the absence of H_2_O_2_ in phosphate buffer (orange line, the conversion refers to 4-(methylthio)phenol) and after the addition of H_2_O_2_ in the presence of 0.0, 0.1, and 1.0 μM r*Aae*UPO. The dashed lines are drawn as a guide for the eye.

In the previous work, we demonstrated the hydrolytic lability of the 4-(methylthio)phenyl ester when the thioether moiety is oxidized to the corresponding sulfoxide.^31^ Here, we synthesized the taurine derivative **1** to study the oxidation process of the thioanisole moiety and whether sulfoxidation triggers carbamate hydrolysis. This model compound was also used to determine the ideal concentration of enzymes to accelerate the oxidation of the thioether unit. To avoid the inactivation of *Ci*VCPO in the presence of inorganic phosphate, we performed all experiments with this chloroperoxidase in citrate buffer (CB, 50 mM, pH = 6.2, 140 mM NaCl). First, we followed product formation from 20 mM **1** in CB upon the addition of 1.5 equiv of H_2_O_2_ in the absence of enzyme (Figure 2A) at 37 °C.

After the first hour, the appearance of low field peaks in ^1^H NMR revealed the formation of sulfoxide **2** and the 4-(methylsulfinyl)phenol **3** (Figure S1). At 3 h, the conversion to **2** and **3** was 55 and 12%, respectively, and 33% of **1** was still present. Compound **1** was completely consumed after 8 h, but the amount of **2** dropped to 45%, while **3** increased to 52%. Sulfoxide **2** is an intermediate that hydrolyzes over time to form **3** and free taurine, demonstrating the hydrolysis of the carbamate moiety in these conditions. It took 16 h to reach more than 80% conversion to phenol **3**, indicating that the hydrolysis rate from **2** to **3** is lower than the rate of oxidation of **1** to **2**. Considering the difficulties in enhancing the rate of hydrolysis, we focused on the catalysis of the oxidation step. Therefore, we tested *Ci*VCPO in concentrations of 0.1 and 1.0 μM to seek for the optimal oxidation conditions of **1**. In the presence of 0.1 μM of *Ci*VCPO, compound **1** converted into 80% of **2** in 2 h after the addition of H_2_O_2_ (Figure 2B, yellow line). With 1.0 μM of *Ci*VCPO, 85% of **2** was produced in 10 min (Figure 2B, purple line). This result demonstrates that the chloroperoxidase accelerates the oxidation of **1** more than two times for 0.1 μM and about 30 times for 1.0 μM enzyme. In addition, at 3 h, the conversion of **3** was 19% for 0.1 μM of *Ci*VCPO and 26% for 1.0 μM of *Ci*VCPO against 12% for the enzyme-free sample (Figure 2C). However, for the samples with the chloroperoxidases, the formation of **3** was about 80% after 12 h. This indicates that the hydrolysis rate is influenced by the concentration of **2**, but this effect fades with the consumption of the sulfoxide and the oxidant, translating into a minor difference in the kinetic profile toward the end of the reaction.

Aiming to investigate the reaction in neutral conditions, we decided to follow the oxidation and hydrolysis of **1** with 1.5 equiv of H_2_O_2_ in phosphate buffer (PB, 50 mM) at pH = 7.0. In Figure 2D, we show the conversion of **1** and its products in PB in the absence of enzyme. At 3 h, the production of **2** and **3** was, respectively, 39 and 28%, with 33% of **1** remaining. At the same time point, **1** was consumed equally whether in phosphate or in citrate buffer, but the higher concentration of **3** in PB indicates that the hydrolysis is indeed faster than in CB. Due to the incompatibility of *Ci*VCPO and phosphate buffer, we employed r*Aae*UPO as a peroxizyme to catalyze the oxidation of **1** for these conditions. Similar to *Ci*VCPO, we used 0.1 and 1.0 μM of r*Aae*UPO to accelerate the oxidation of **1** with H_2_O_2_. Compound **2** reached 90% in only 10 min with 1.0 μM of r*Aae*UPO and 48% in 2 h with 0.1 μM of r*Aae*UPO (Figure 2E). The hydrolysis profile of **2** to **3** (Figure 2F) was similar in all cases, reaching about 90% in 12 h. The use of 0.1 μM of r*Aae*UPO barely accelerated the oxidation of **1** compared to the uncatalyzed case. In the presence of 1.0 μM of r*Aae*UPO, we have an almost immediate full conversion to **2**, similar to the use of the same concentration of *Ci*VCPO. Finally, at 12 h, the hydrolysis rate reached 90% in PB against 80% in CB. Additionally, after 12 h in the absence of oxidant, 14% of 4-(methylthio)phenol had formed in PB (orange line, Figure 2F) against about 5% in CB (orange line, Figure 2C), confirming the former condition as more advantageous for hydrolytic degradation. Considering the comparable efficiency of both *Ci*VCPO and r*Aae*UPO at a concentration of 1.0 μM in the H_2_O_2_-driven oxidation of the thioether moiety and the balance between responsiveness and stability of the nonoxidized substrate, we chose to explore these conditions at the material level in both PB and CB with the corresponding 1.0 μM enzyme.

### Properties of MTpcFF Hydrogels

Short peptide-based amphiphiles have great potential to form gels encapsulating large amounts of water.^52,53^ Considering its promising gelation properties,^54^ we chose **FF** as a peptide building block and functionalized its N-terminus with the 4-(methylthio)phenyl moiety to obtain **MTpcFF**. Using the tube-inversion method, we found that this dipeptide derivative has a critical gel concentration (CGC) of 0.70 wt % in CB (Figure S5) and 0.95 wt % in PB (Figure S6).

Knowing that tripeptide-based hydrogelators have generally lower CGC, we also synthesized **MTpcFFF**. However, in the tube-inversion tests, the gelation of this derived tripeptide was often difficult to achieve and replicate. We indeed noticed poor solubility in aqueous solution even at a concentration of 0.1 wt %, and without the possibility of heating to avoid the premature hydrolysis of the carbamate motif, we decided to continue our studies exclusively on **MTpcFF**.

**Figure 3:**
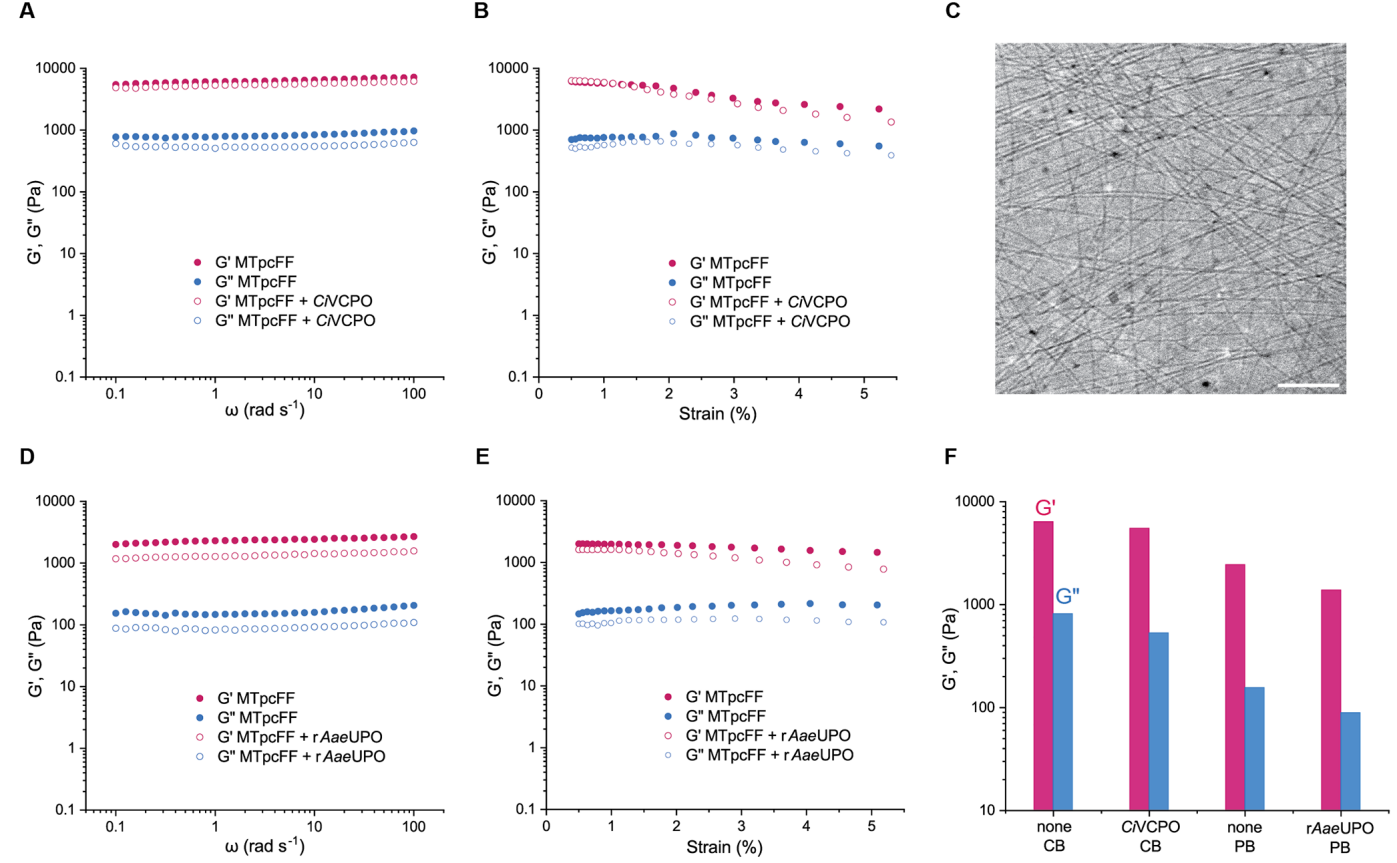
Properties of **MTpcFF** gels. (A) Frequency sweep and (B) strain sweep rheological properties of **MTpcFF** with (open circles) and without (filled circles) *Ci*VCPO in citrate buffer. (C) Cryo-EM image of **MTpcFF** (1.0 wt %) hydrogel in PB (scale bar = 200 nm). (D) Frequency sweep and (E) strain sweep rheological properties of **MTpcFF** with (open circles) and without (filled circles) r*Aae*UPO in phosphate buffer. (F) Rheological properties of **MTpcFF** gels at an angular frequency of 1.0 Hz.

First, we investigated the rheological properties of **MTpcFF** at 0.75 wt % in CB and at 1.0 wt % in PB. Interestingly, despite the higher concentration of the hydrogelator in PB, the storage modulus (*G*′) is 2.5 kPa and the loss modulus (*G*″) is 0.2 kPa for **MTpcFF** in PB against 6.4 (*G*′) and 0.8 (*G*″) kPa for **MTpcFF** in CB (Figure 3).

This result is not surprising if we consider the influence that pH and Hofmeister effect have over the gelation of other phenylalanine-based LMWG.^55−60^ Lower pH leads to a higher degree of protonation of the terminal carboxylic acid of the dipeptide amphiphile, making the functionalized **FF** more hydrophobic and therefore favoring the gelation in aqueous solution. Nevertheless, obtaining *G*′ values always higher than the *G*″ means that both conditions produced gels with viscoelastic properties typical of fiber networks.^61^ Observation of the hydrogels with optical microscopy (Figure S3) and cryo-EM images (Figures 3C and S4) confirmed the presence of nanofibers that can aggregate in bundles with microscopic diameters. Analysis of cryo-EM images showed average fiber diameters of 7.9 ± 2.0 (citrate buffer, Figure S4C) and 4.0 ± 0.7 nm (phosphate buffer, Figure S4F) for the **MTpcFF** hydrogels.

Additives and impurities can affect the gelation of LMWG. Thus, to exclude that the encapsulation of *Ci*VCPO and r*Aae*UPO influences the mechanical properties of **MTpcFF** gels, we performed additional rheological analysis on the enzyme-loaded hydrogels. The resulting data confirmed that adding 1.0 μM of *Ci*VCPO to 0.75 wt % of **MTpcFF** in CB and 1.0 μM of r*Aae*UPO to 1.0 wt % of **MTpcFF** in PB produced *G*′ and *G*″ values that are only slightly lower than the corresponding gels without peroxizymes (Figure 3F). We also performed strain amplitude measurements within the linear viscoelastic region for all four different conditions. **MTpcFF** gels in CB both with and without *Ci*VCPO showed a slight drop in *G*′ and *G*″ values at 3% strain. In contrast, despite their lower storage and loss moduli, **MTpcFF** gels in PB demonstrated good stability even at 5% strain. Knowing these minor differences in the mechanical properties of **MTpcFF** hydrogel in different conditions, we pursued to investigate their response to H_2_O_2_.

### H_2_O_2_-Responsive Gel–Sol Transition of MTpcFF Hydrogels

**Figure 4:**
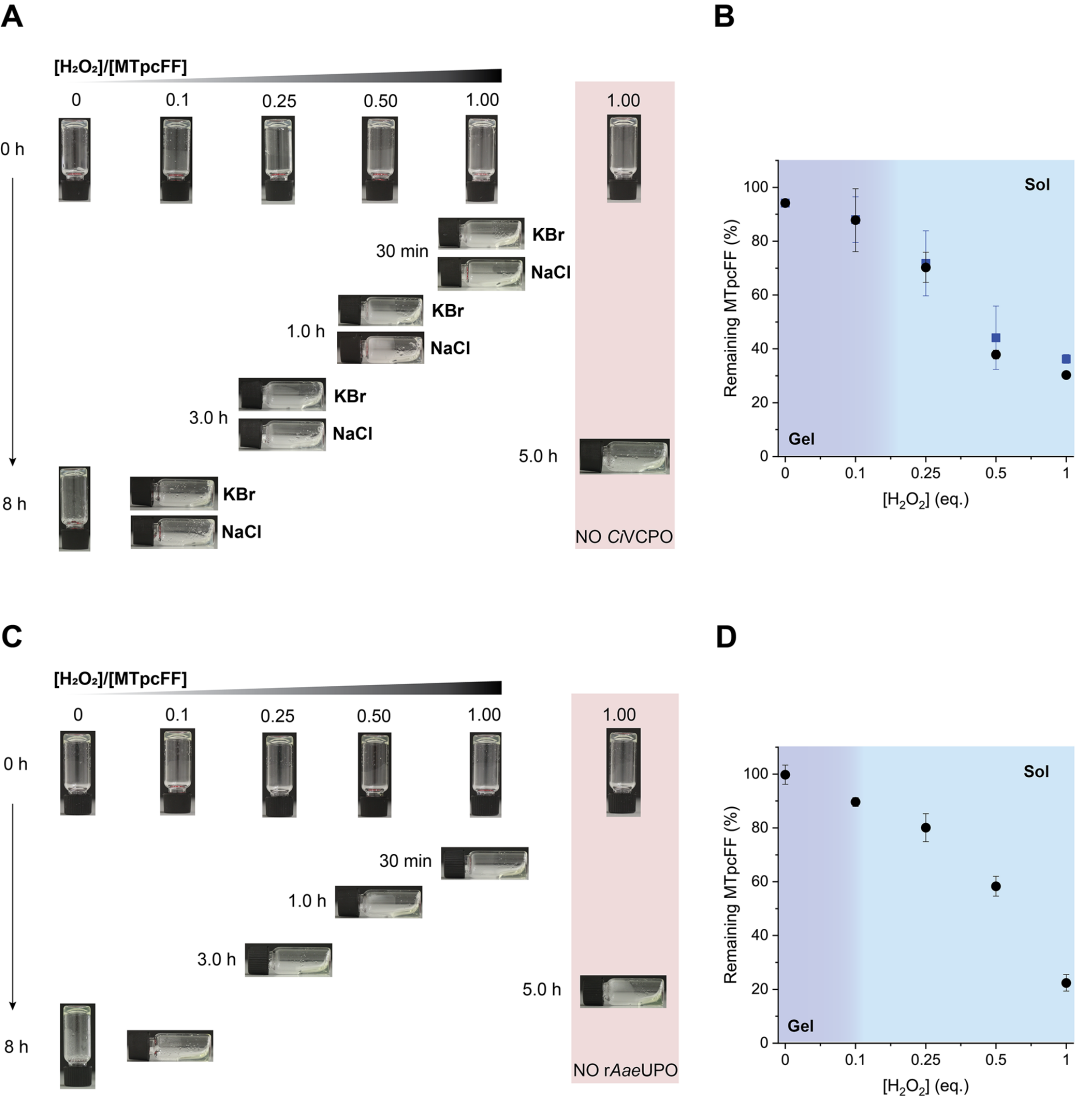
(A) Photographs of *Ci*VCPO (1.0 μM)-loaded **MTpcFF** hydrogels in CB after the addition of various amounts of H_2_O_2_. (B) HPLC analysis of the remaining **MTpcFF** after the addition of various amounts of H_2_O_2_ in the presence of *Ci*VCPO (1.0 μM) in CB with NaCl (filled circles) or KBr (filled squares). The experiments were performed in duplicate to obtain the mean and standard deviation values (shown as error bars). (C) Photographs of r*Aae*UPO (1.0 μM)-loaded **MTpcFF** hydrogels in PB after the addition of various amounts of H_2_O_2_. (D) HPLC analysis of the remaining **MTpcFF** after the addition of various amounts of H_2_O_2_ in the presence of r*Aae*UPO (1.0 μM) in PB. The experiments were performed in duplicate to obtain the mean and standard deviation values (shown as error bars).

To test the sensitivity of **MTpcFF** gels toward oxidation, we followed the gel–sol transition in time after the addition of H_2_O_2_ via the tube-inversion method. **MTpcFF** hydrogels (0.75 wt %, 16 mM) were prepared in CB with and without *Ci*VCPO (1.0 μM) and visually followed after the addition of varying equivalents of H_2_O_2_ (Figure 4A).

The peroxizyme-free gels turned into solution 5 h after the addition of 1.0 equiv of H_2_O_2_, while *Ci*VCPO-loaded **MTpcFF** hydrogels became solutions within 30 min in the presence of an equimolar amount of hydrogen peroxide (Figure 4A). The hydrogels with *Ci*VCPO fully disrupted in 1 and 3 h with 0.5 and 0.25 equiv of H_2_O_2_, respectively. Meanwhile, the *Ci*VCPO-loaded control gels, where the oxidant was not added, remained intact for over 8 h.

To investigate the molecular mechanism behind the material change, we analyzed **MTpcFF** hydrogels as soon as they had disintegrated after the addition of 1.0 equiv. of H_2_O_2_ with the high-performance liquid chromatography (HPLC). In the chromatogram, we found that after this time the peak of **MTpcFF** has decreased, while an unidentified peak appeared at *t*_R_ = 18.7 min. LC-MS analysis revealed that we obtained the corresponding sulfoxide of the gelator (Figure S9). Despite the presence in the HPLC analysis of about 15% **FF** 5 h after the addition of H_2_O_2_, we concluded that the main cause of the disassembly of the hydrogel is the oxidation of **MTpcFF** into the more hydrophilic sulfoxide. This result comes unexpected since the sulfoxidation-related change in hydrophilicity was previously considered to be too small to lead to the disassembly of block copolymers based on this motif.^31^ On the other hand, the observed gel disruption at the oxidation step accelerates the material response and removes the slow hydrolysis as the rate-determining step. In addition, the slow hydrolysis rate explains the considerable stability of the hydrogels in the absence of the oxidant.

The 10-fold acceleration of gel–sol transition encouraged us to explore [H_2_O_2_]/[**MTpcFF**] ratios as low as 0.1. Interestingly, **MTpcFF** hydrogels were responsive to such low concentrations of H_2_O_2_, even if the gel solubilization was not complete at 8 h (Figure 4A, bottom left). The HPLC analysis was performed on the solutions obtained after 5 h for the hydrogels exposed to high oxidant concentration, while the partially disrupted gels exposed to 0.1 equiv of H_2_O_2_ and the control experiments were analyzed after 8 h. The HPLC data in Figure 4B show that the presence of 0.1 equiv of H_2_O_2_ caused the consumption of about 10% gelator. Considering the corresponding gel picture, we can conclude that this variation in the **MTpcFF** concentration, and therefore the addition of 0.1 equiv of H_2_O_2_, is not sufficient to completely degrade the gel. In contrast, upon the addition of 0.25 equiv of H_2_O_2_ we obtained full collapse of the gels in 3 h, with about 75% of the remaining gelator according to HPLC analysis. This suggests that the threshold for full solubilization of an **MTpcFF** gel (0.75 wt %) in CB is in the range of 0.1–0.2 equiv of H_2_O_2_. All of these experiments were carried out either with hydrogen peroxide and either NaCl or KBr, needed for the production of hypohalites by *Ci*VCPO. The chloroperoxidase is able to convert H_2_O_2_ and NaCl into HOCl, while in the presence of KBr, HOBr is formed. In principle, HOCl has a higher oxidation potential than HOBr, while the latter is more electrophilic and can react faster with the thioether.^62^ In the tube-inversion tests, the use of NaCl together with H_2_O_2_ led to solutions that appeared slightly less viscous than when KBr was used. In the correlating HPLC analysis, for additions of 1.0 and 0.5 equiv of H_2_O_2_, about 6% less **MTpcFF** was measured when NaCl was added instead of KBr. However, the difference is not significant, making it difficult to assess what species has more impact on the system. On a second note, considering the abundant presence of chlorine in the cellular environment and the tendency of HOBr to react with a broad range of substrates other than the thioether,^63,64^ the conditions with NaCl should be favorable for biological applications.

Subsequently, we proceeded to test the gel disruption upon the addition of H_2_O_2_ between 0.1 and 1.0 equiv on 1.0 wt % of **MTpcFF** hydrogels (20 mM) in PB with and without r*Aae*UPO (1.0 μM). The photographs of these inverted vial tests are shown in Figure 4C. r*Aae*UPO-free hydrogels take 5 h to turn into a viscous solution in the presence of 1.0 equiv of the oxidant, while when r*Aae*UPO was encapsulated in the gel formulation, this time reduced to 30 min. The time to gel degradation was 1 and 3 h when, respectively, 0.5 and 0.25 equiv of H_2_O_2_ were added. We found that 0.1 equiv of hydrogen peroxide is enough to achieve the gel–sol transition even if the time scale is extended to 8 h in this case. The corresponding HPLC results (Figure 4D) showed that for 0.1 equiv of H_2_O_2_, the expected 90% of **MTpcFF** was detected, which appeared to be below the CGC of the gels when compared with the tube-inversion tests. Such a low threshold is in line with the need for increasing the gelator concentration when performing gelation in PB compared to that achieved in CB. Additionally, we tested the stability of previously formed **MTpcFF** hydrogels in both PB and CB solutions, showing that **MTpcFF** hydrogels are stable in 500 μL of citrate buffer for 20 h and in 200 μL of phosphate buffer for more than 8 h (Figure S7). These results demonstrate good stability of **MTpcFF** hydrogels compared to their responsiveness toward H_2_O_2_.

Despite the different concentrations and conditions of **MTpcFF** hydrogels with r*Aae*UPO and with *Ci*VCPO, the response times in gel collapse upon the addition of H_2_O_2_ were remarkably similar. The close catalytic effect of 1.0 μM of r*Aae*UPO and of 1.0 μM of *Ci*VCPO (in the presence of halides) to oxidize the thioether with H_2_O_2_ was already anticipated from the ^1^H NMR study on **1** (Figure 2). The translation of this effect to the hydrogels with different formulations enables these materials to have comparable sensitivity toward H_2_O_2_ (∼2.0 mM), in both mildly acidic and neutral settings.

## Conclusions

This study presents a thioether carbamate-based dipeptide that can form stable hydrogels in the pH range of 6.0–7.0. The addition of H_2_O_2_ leads to oxidation of the thioether to sulfoxide, triggering a solubility switch and disruption of the gel. We encapsulated two different peroxizymes in the hydrogel: the vanadium-dependent chloroperoxidase *Ci*VCPO in citrate buffer at pH 6.2 and the heme-dependent peroxidase r*Aae*UPO in phosphate buffer at pH 7.0. Enzymatic hydrogen peroxide activation in both cases resulted in the gel collapsing 10 times faster than peroxizyme-free samples. The encapsulation of peroxizymes in the gel matrix proved essential to achieve a gel–sol transition at hydrogen peroxide concentrations below 2.0 mM. Moreover, we believe that the similar responsiveness of the hydrogel in two different conditions is a key feature for application of this system in neutral and mildly acidic environments with elevated ROS concentrations.

This simple dipeptide-based gelator could be used to create a library of thioanisole carbamate-based LMWGs with different mechanical properties and response thresholds, making it a promising material for devices sensitive to redox imbalance in biological settings. Additionally, although not essential in this study, the hydrolytic instability of the carbamate after oxidation makes the thioether phenyl group a potential ROS-labile protecting group for amines in pharmaceutically active compounds.

## Supporting Information Available

Methods description; additional synthetic and characterization procedures; ^1^H NMR and ^13^C NMR spectra of the synthesized molecular models and hydrogelators; hydrogel characterization procedures; photographs of the inverted tube tests; optical microscopy and cryo-EM images of the hydrogels, and frequency distribution of the gel fiber diameters and HPLC analysis (PDF)

## References

[ref1] Ni M.; Zhuo S. Applications of self-assembling ultrashort peptides in bionanotechnology. RSC Adv. 2019, 9, 844–852. 10.1039/C8RA07533F. 35517614 PMC9059570

[ref2] Yadav N.; Chauhan M. K.; Chauhan V. S. Short to ultrashort peptide-based hydrogels as a platform for biomedical applications. Biomater. Sci. 2020, 8, 84–100. 10.1039/C9BM01304K. 31696870

[ref3] Weiss R. G. The Past, Present, and Future of Molecular Gels. What Is the Status of the Field, and Where Is It Going?. J. Am. Chem. Soc. 2014, 136, 7519–7530. 10.1021/ja503363v. 24836858

[ref4] Gupta J. K.; Adams D. J.; Berry N. G. Will it gel? Successful computational prediction of peptide gelators using physicochemical properties and molecular fingerprints. Chem. Sci. 2016, 7, 4713–4719. 10.1039/C6SC00722H. 30155120 PMC6016447

[ref5] Mendes A. C.; Baran E. T.; Reis R. L.; Azevedo H. S. Self-assembly in nature: using the principles of nature to create complex nanobiomaterials. Wiley Interdiscip. Rev.: Nanomed. Nanobiotechnol. 2013, 5, 582–612. 10.1002/wnan.1238. 23929805

[ref6] Vegners R.; Shestakova I.; Kalvinsh I.; Ezzell R. M.; Janmey P. A. Use of a gel-forming dipeptide derivative as a carrier for antigen presentation. J. Pept. Sci. 1995, 1, 371–378. 10.1002/psc.310010604. 9223016

[ref7] Reches M.; Gazit E. Casting Metal Nanowires Within Discrete Self-Assembled Peptide Nanotubes. Science 2003, 300, 625–627. 10.1126/science.1082387. 12714741

[ref8] Roytman R.; Adler-Abramovich L.; Kumar K. S. A.; Kuan T.-C.; Lin C.-C.; Gazit E.; Brik A. Exploring the self-assembly of glycopeptides using a diphenylalanine scaffold. Org. Biomol. Chem. 2011, 9, 5755–5761. 10.1039/c1ob05071k. 21720631

[ref9] Krysmann M. J.; Castelletto V.; Kelarakis A.; Hamley I. W.; Hule R. A.; Pochan D. J. Self-Assembly and Hydrogelation of an Amyloid Peptide Fragment. Biochemistry 2008, 47, 4597–4605. 10.1021/bi8000616. 18370402

[ref10] Kim J.; Han T. H.; Kim Y.-I.; Park J. S.; Choi J.; Churchill D. G.; Kim S. O.; Ihee H. Role of Water in Directing Diphenylalanine Assembly into Nanotubes and Nanowires. Adv. Mater. 2010, 22, 583–587. 10.1002/adma.200901973. 20217753

[ref11] Görbitz C. H. The structure of nanotubes formed by diphenylalanine, the core recognition motif of Alzheimer’s β-amyloid polypeptide. Chem. Commun. 2006, 22, 2332–2334. 10.1039/B603080G. 16733570

[ref12] Kuang Y.; Du X.; Zhou J.; Xu B. Supramolecular Nanofibrils Inhibit Cancer Progression In Vitro and In Vivo. Adv. Healthcare Mater. 2014, 3, 1217–1221. 10.1002/adhm.201300645. PMC4134730 24574174

[ref13] Ischakov R.; Adler-Abramovich L.; Buzhansky L.; Shekhter T.; Gazit E. Peptide-based hydrogel nanoparticles as effective drug delivery agents. Biorg. Med. Chem. 2013, 21, 3517–3522. 10.1016/j.bmc.2013.03.012. 23566763

[ref14] Diaferia C.; Morelli G.; Accardo A. Fmoc-diphenylalanine as a suitable building block for the preparation of hybrid materials and their potential applications. J. Mater. Chem. B 2019, 7, 5142–5155. 10.1039/C9TB01043B. 31380554

[ref15] Cardoso A. Z.; Alvarez A. E. A.; Cattoz B. N.; Griffiths P. C.; King S. M.; Frith W. J.; Adams D. J. The influence of the kinetics of self-assembly on the properties of dipeptide hydrogels. Faraday Discuss. 2013, 166, 101–116. 10.1039/c3fd00104k. 24611271

[ref16] Tang C.; Ulijn R. V.; Saiani A. Effect of Glycine Substitution on Fmoc–Diphenylalanine Self-Assembly and Gelation Properties. Langmuir 2011, 27, 14438–14449. 10.1021/la202113j. 21995651

[ref17] Yang Z.; Liang G.; Wang L.; Xu B. Using a Kinase/Phosphatase Switch to Regulate a Supramolecular Hydrogel and Forming the Supramolecular Hydrogel in Vivo. J. Am. Chem. Soc. 2006, 128, 3038–3043. 10.1021/ja057412y. 16506785

[ref18] Yi M.; Guo J.; He H.; Tan W.; Harmon N.; Ghebreyessus K.; Xu B. Phosphobisaromatic motifs enable rapid enzymatic self-assembly and hydrogelation of short peptides. Soft Matter 2021, 17, 8590–8594. 10.1039/D1SM01221E. 34545895 PMC8600407

[ref19] Huang Y.; Qiu Z.; Xu Y.; Shi J.; Lin H.; Zhang Y. Supramolecular hydrogels based on short peptides linked with conformational switch. Org. Biomol. Chem. 2011, 9, 2149–2155. 10.1039/c0ob01057j. 21298187

[ref20] Sahoo J. K.; Nalluri S. K. M.; Javid N.; Webb H.; Ulijn R. V. Biocatalytic amide condensation and gelation controlled by light. Chem. Commun. 2014, 50, 5462–5464. 10.1039/C4CC01431F. 24714972

[ref21] Ikeda M.; Tanida T.; Yoshii T.; Hamachi I. Rational Molecular Design of Stimulus-Responsive Supramolecular Hydrogels Based on Dipeptides. Adv. Mater. 2011, 23, 2819–2822. 10.1002/adma.201004658. 21538587

[ref22] Ikeda M.; Tanida T.; Yoshii T.; Kurotani K.; Onogi S.; Urayama K.; Hamachi I. Installing logic-gate responses to a variety of biological substances in supramolecular hydrogel–enzyme hybrids. Nat. Chem. 2014, 6, 511–518. 10.1038/nchem.1937. 24848237

[ref23] Napoli A.; Valentini M.; Tirelli N.; Müller M.; Hubbell J. A. Oxidation-responsive polymeric vesicles. Nat. Mater. 2004, 3, 183–189. 10.1038/nmat1081. 14991021

[ref24] El Mohtadi F.; d’Arcy R.; Burke J.; De La Rosa J. M. R.; Gennari A.; Marotta R.; Francini N.; Donno R.; Tirelli N. “Tandem” Nanomedicine Approach against Osteoclastogenesis: Polysulfide Micelles Synergically Scavenge ROS and Release Rapamycin. Biomacromolecules 2020, 21, 305–318. 10.1021/acs.biomac.9b01348. 31793790

[ref25] Geven M.; d’Arcy R.; Turhan Z. Y.; El-Mohtadi F.; Alshamsan A.; Tirelli N. Sulfur-based oxidation-responsive polymers. Chemistry, (chemically selective) responsiveness and biomedical applications. Eur. Polym. J. 2021, 149, 110387 10.1016/j.eurpolymj.2021.110387.

[ref26] Yu S.; Wang C.; Yu J.; Wang J.; Lu Y.; Zhang Y.; Zhang X.; Hu Q.; Sun W.; He C.; Chen X.; Gu Z. Injectable Bioresponsive Gel Depot for Enhanced Immune Checkpoint Blockade. Adv. Mater. 2018, 30, 1801527 10.1002/adma.201801527. 29786888

[ref27] Xu Q.; He C.; Ren K.; Xiao C.; Chen X. Thermosensitive Polypeptide Hydrogels as a Platform for ROS-Triggered Cargo Release with Innate Cytoprotective Ability under Oxidative Stress. Adv. Healthcare Mater. 2016, 5, 1979–1990. 10.1002/adhm.201600292. 27283999

[ref28] Spitzer D.; Rodrigues L. L.; Straßburger D.; Mezger M.; Besenius P. Tuneable Transient Thermogels Mediated by a pH- and Redox-Regulated Supramolecular Polymerization. Angew. Chem., Int. Ed. 2017, 56, 15461–15465. 10.1002/anie.201708857. 29044991

[ref29] Miao X.; Cao W.; Zheng W.; Wang J.; Zhang X.; Gao J.; Yang C.; Kong D.; Xu H.; Wang L.; Yang Z. Switchable Catalytic Activity: Selenium-Containing Peptides with Redox-Controllable Self-Assembly Properties. Angew. Chem., Int. Ed. 2013, 52, 7781–7785. 10.1002/anie.201303199. 23784972

[ref30] Criado-Gonzalez M.; Mecerreyes D. Thioether-based ROS responsive polymers for biomedical applications. J. Mater. Chem. B 2022, 10, 7206–7221. 10.1039/D2TB00615D. 35611805

[ref31] Piergentili I.; Bouwmans P. R.; Reinalda L.; Lewis R. W.; Klemm B.; Liu H.; de Kruijff R. M.; Denkova A. G.; Eelkema R. Thioanisole ester based logic gate cascade to control ROS-triggered micellar degradation. Polym. Chem. 2022, 13, 2383–2390. 10.1039/D2PY00207H. 35664499 PMC9016795

[ref32] Allen B. L.; Johnson J. D.; Walker J. P. Encapsulation and Enzyme-Mediated Release of Molecular Cargo in Polysulfide Nanoparticles. ACS Nano 2011, 5, 5263–5272. 10.1021/nn201477y. 21595444

[ref33] Tóth Z.; Fábián I. Oxidation of Chlorine(III) by Hypobromous Acid: Kinetics and Mechanism. Inorg. Chem. 2004, 43, 2717–2723. 10.1021/ic0354318. 15074992

[ref34] Hu Y.; Xie G.; Stanbury D. M. Oxidations at Sulfur Centers by Aqueous Hypochlorous Acid and Hypochlorite: Cl+ Versus O Atom Transfer. Inorg. Chem. 2017, 56, 4047–4056. 10.1021/acs.inorgchem.6b03182. 28290673

[ref35] Höfler G. T.; But A.; Hollmann F. Haloperoxidases as catalysts in organic synthesis. Org. Biomol. Chem. 2019, 17, 9267–9274. 10.1039/C9OB01884K. 31599911

[ref36] Fernández-Fueyo E.; van Wingerden M.; Renirie R.; Wever R.; Ni Y.; Holtmann D.; Hollmann F. Chemoenzymatic Halogenation of Phenols by using the Haloperoxidase from *Curvularia inaequalis*. ChemCatChem 2015, 7, 4035–4038. 10.1002/cctc.201500862.

[ref37] Fernández-Fueyo E.; Younes S. H. H.; van Rootselaar S.; Aben R. W. M.; Renirie R.; Wever R.; Holtmann D.; Rutjes F. P. J. T.; Hollmann F. A Biocatalytic Aza-Achmatowicz Reaction. ACS Catal. 2016, 6, 5904–5907. 10.1021/acscatal.6b01636.

[ref38] Dong J. J.; Fernández-Fueyo E.; Li J.; Guo Z.; Renirie R.; Wever R.; Hollmann F. Halofunctionalization of alkenes by vanadium chloroperoxidase from *Curvularia inaequalis*. Chem. Commun. 2017, 53, 6207–6210. 10.1039/C7CC03368K. 28548142

[ref39] Wever R.; Renirie R.; Hollmann F. Vanadium Chloroperoxidases as Versatile Biocatalysts. In Vanadium Catalysis; Sutradhar M.; Pombeiro A. J. L.; da Silva J. A. L., Eds.; The Royal Society of Chemistry, 2021; pp 548–563.

[ref40] Lin B.; Chen H.; Liang D.; Lin W.; Qi X.; Liu H.; Deng X. Acidic pH and High-H2O2 Dual Tumor Microenvironment-Responsive Nanocatalytic Graphene Oxide for Cancer Selective Therapy and Recognition. ACS Appl. Mater. Interfaces 2019, 11, 11157–11166. 10.1021/acsami.8b22487. 30869853

[ref41] Ozaki S.-i.; Yang H.-J.; Matsui T.; Goto Y.; Watanabe Y. Asymmetric oxidation catalyzed by myoglobin mutants. Tetrahedron: Asymmetry 1999, 10, 183–192. 10.1016/S0957-4166(98)00498-4.

[ref42] Baciocchi E.; Gerini M. F.; Harvey P. J.; Lanzalunga O.; Mancinelli S. Oxidation of aromatic sulfides by lignin peroxidase from Phanerochaete chrysosporium. Eur. J. Biochem. 2000, 267, 2705–2710. 10.1046/j.1432-1327.2000.01293.x. 10785393

[ref43] Peter S.; Kinne M.; Wang X.; Ullrich R.; Kayser G.; Groves J. T.; Hofrichter M. Selective hydroxylation of alkanes by an extracellular fungal peroxygenase. FEBS J. 2011, 278, 3667–3675. 10.1111/j.1742-4658.2011.08285.x. 21812933 PMC3586278

[ref44] Kluge M.; Ullrich R.; Dolge C.; Scheibner K.; Hofrichter M. Hydroxylation of naphthalene by aromatic peroxygenase from *Agrocybe aegerita* proceeds via oxygen transfer from H2O2 and intermediary epoxidation. Appl. Microbiol. Biotechnol. 2009, 81, 1071–1076. 10.1007/s00253-008-1704-y. 18815784

[ref45] Höfler G. T.; But A.; Younes S. H. H.; Wever R.; Paul C. E.; Arends I. W. C. E.; Hollmann F. Chemoenzymatic Halocyclization of 4-Pentenoic Acid at Preparative Scale. ACS Sustainable Chem. Eng. 2020, 8, 2602–2607. 10.1021/acssuschemeng.9b07494. 32117647 PMC7045808

[ref46] Zippilli C.; Bartolome M. J.; Hilberath T.; Botta L.; Hollmann F.; Saladino R. A Photochemoenzymatic Hunsdiecker-Borodin-Type Halodecarboxylation of Ferulic Acid. ChemBioChem 2022, 23, e202200367 10.1002/cbic.202200367. 35921215 PMC9804872

[ref47] Tonin F.; Tieves F.; Willot S.; van Troost A.; van Oosten R.; Breestraat S.; van Pelt S.; Alcalde M.; Hollmann F. Pilot-Scale Production of Peroxygenase from *Agrocybe aegerita*. Org. Process Res. Dev. 2021, 25, 1414–1418. 10.1021/acs.oprd.1c00116. 34168423 PMC8218300

[ref48] Molina-Espeja P.; Garcia-Ruiz E.; Gonzalez-Perez D.; Ullrich R.; Hofrichter M.; Alcalde M. Directed Evolution of Unspecific Peroxygenase from *Agrocybe aegerita*. Appl. Environ. Microbiol. 2014, 80, 3496–3507. 10.1128/AEM.00490-14. 24682297 PMC4018863

[ref49] Freer R.; McKillop A. Synthesis of Symmetrical and Unsymmetrical Ureas Using Unsymmetrical Diaryl Carbonates. Synth. Commun. 1996, 26, 331–349. 10.1080/00397919608003622.

[ref50] Dadhwal S.; Fairhall J. M.; Goswami S. K.; Hook S.; Gamble A. B. Alkene–Azide 1,3-Dipolar Cycloaddition as a Trigger for Ultrashort Peptide Hydrogel Dissolution. Chem. - Asian J. 2019, 14, 1143–1150. 10.1002/asia.201801184. 30324726

[ref51] Tanwar D. K.; Ratan A.; Gill M. S. A facile synthesis of sulfonylureas via water assisted preparation of carbamates. Org. Biomol. Chem. 2017, 15, 4992–4999. 10.1039/C7OB00872D. 28567464

[ref52] de Loos M.; Feringa B. L.; van Esch J. H. Design and Application of Self-Assembled Low Molecular Weight Hydrogels. Eur. J. Org. Chem. 2005, 2005, 3615–3631. 10.1002/ejoc.200400723.

[ref53] Estroff L. A.; Hamilton A. D. Water Gelation by Small Organic Molecules. Chem. Rev. 2004, 104, 1201–1218. 10.1021/cr0302049. 15008620

[ref54] Adler-Abramovich L.; Vaks L.; Carny O.; Trudler D.; Magno A.; Caflisch A.; Frenkel D.; Gazit E. Phenylalanine assembly into toxic fibrils suggests amyloid etiology in phenylketonuria. Nat. Chem. Biol. 2012, 8, 701–706. 10.1038/nchembio.1002. 22706200

[ref55] Raeburn J.; Pont G.; Chen L.; Cesbron Y.; Lévy R.; Adams D. J. Fmoc-diphenylalanine hydrogels: understanding the variability in reported mechanical properties. Soft Matter 2012, 8, 1168–1174. 10.1039/C1SM06929B. 36345210

[ref56] Fleming S.; Ulijn R. V. Design of nanostructures based on aromatic peptide amphiphiles. Chem. Soc. Rev. 2014, 43, 8150–8177. 10.1039/C4CS00247D. 25199102

[ref57] Hofmeister F. Zur Lehre von der Wirkung der Salze. Arch. Exp. Pathol. Pharmakol. 1888, 24, 247–260. 10.1007/BF01918191.

[ref58] Roy S.; Javid N.; Frederix P. W. J. M.; Lamprou D. A.; Urquhart A. J.; Hunt N. T.; Halling P. J.; Ulijn R. V. Dramatic Specific-Ion Effect in Supramolecular Hydrogels. Chem. - Eur. J. 2012, 18, 11723–11731. 10.1002/chem.201201217. 22888053

[ref59] Gregory K. P.; Elliott G. R.; Robertson H.; Kumar A.; Wanless E. J.; Webber G. B.; Craig V. S. J.; Andersson G. G.; Page A. J. Understanding specific ion effects and the Hofmeister series. Phys. Chem. Chem. Phys. 2022, 24, 12682–12718. 10.1039/D2CP00847E. 35543205

[ref60] Abraham B. L.; Agredo P.; Mensah S. G.; Nilsson B. L. Anion Effects on the Supramolecular Self-Assembly of Cationic Phenylalanine Derivatives. Langmuir 2022, 38, 15494–15505. 10.1021/acs.langmuir.2c01394. 36473193 PMC9776537

[ref61] Aggeli A.; Bell M.; Boden N.; Keen J. N.; Knowles P. F.; McLeish T. C. B.; Pitkeathly M.; Radford S. E. Responsive gels formed by the spontaneous self-assembly of peptides into polymeric β-sheet tapes. Nature 1997, 386, 259–262. 10.1038/386259a0. 9069283

[ref62] Ximenes V. F.; Morgon N. H.; de Souza A. R. Hypobromous acid, a powerful endogenous electrophile: Experimental and theoretical studies. J. Inorg. Biochem. 2015, 146, 61–68. 10.1016/j.jinorgbio.2015.02.014. 25771434

[ref63] Hawkins C. L.; Davies M. J. Role of myeloperoxidase and oxidant formation in the extracellular environment in inflammation-induced tissue damage. Free Radical Biol. Med. 2021, 172, 633–651. 10.1016/j.freeradbiomed.2021.07.007. 34246778

[ref64] Davies M. J. Myeloperoxidase-derived oxidation: mechanisms of biological damage and its prevention. J. Clin. Biochem. Nutr. 2010, 48, 8–19. 10.3164/jcbn.11-006FR. 21297906 PMC3022070

