# Supplementary material for: Enhancing the ROS Sensitivity of a Responsive Supramolecular Hydrogel Using Peroxizyme Catalysis
Source: Biomacromolecules. 2023 Jun 23;24(7):3184–92. doi: 10.1021/acs.biomac.3c00262 (PMC10336921; doi:10.1021/acs.biomac.3c00262)
Supplement: Supplementary file 1 — bm3c00262_si_001.pdf [file bm3c00262_si_001.pdf]

Supporting Information

Enhancing the ROS Sensitivity of a Responsive Supramolecular Hydrogel using Peroxizyme Catalysis

Irene Piergentili[a], Thomas Hilberath[b], Benjamin Klemm[a], Frank Hollmann[b], Rienk Eelkema[a*]

[a] Delft University of Technology, Department of Chemical Engineering, Van der Maasweg 9, 2629 HZ Delft, The Netherlands

[b] Delft University of Technology, Department of Biotechnology, Van der Maasweg 9, 2629 HZ Delft, The Netherlands

* correspondence to r.eelkema@tudelft.nl

Supplementary methods

^1H NMR and ^{13}C NMR spectra were recorded on Agilent-400 MR DD2 (400 MHz and 100.5 MHz for ^1H and ^{13}C, respectively) spectrometer at 298 K. Chemical shifts are reported in ppm relative to the residual solvent peak, the multiplicity is reported as follows: s = singlet, d = doublet, t = triplet, q = quartet, m = multiplet, and J-couplings (*J*) are reported in Hertz (Hz). ESI-MS was performed using LTQ XL spectrometer equipped with Shimadzu HPLC setup operating at 0.2 mL/min flow rate with water/MeCN mobile phase containing 0.1 vol% formic acid and Discovery C18 column. Reactions were monitored by analytical thin-layer chromatography (TLC) on silica gel plates (Merck 60 F_{254}) using UV light (254 nm) as the visualizing agent. Flash chromatography was performed on 230-400 mesh silica gel (Sigma Aldrich). Reverse phase HPLC (RP-HPLC) for compound purification was performed with a Shimadzu LC-20 system with a Shimadzu mSPD-20A Photo Diode Array detector. RP-HPLC of the collapsed gels was conducted with a Waters Acquity UPLC instrument. Photographs of the hydrogels were taken on a Canon EOS 600D single reflex camera with a Canon Macro Lens EF 100 mm 1:2.8 USM. Optical microscopy images were obtained via an inverted microscope (Zeiss Axio Observer) equipped with a 20x objective (N-Achroplan 20x/0.45 M27) and a CCD camera (Axiocam 705 color). Cryo-EM images were aquired on a Jeol JEM1400 plus Transmission Electron Microscope with an operating voltage of 120 kV and TVIPS F416 camera.

Synthesis of MTpcFFF

To a solution of 4-(methylthio)phenyl 4-nitrophenyl carbonate (0.18 g, 0.60 mmol) in THF (16.0 mL) was added dropwise FFF (0.33 g, 0.72 mmol) and DIPEA (0.12 mL, 0.72 mmol) in distilled water (4.0 mL) at 0 °C. The reaction mixture was allowed to warm at room temperature and stirred overnight.[1, 2] TLC analysis confirmed that no 4-(methylthio)phenyl 4-nitrophenyl carbonate remained. After removal of THF through rotatory evaporation, the aqueous mixture was acidified (pH = 2-3) with 5% citric acid solution. The reaction mixture was extracted with ethyl acetate (3x40 mL) and the combined organic layer was washed with water. The organic layer was dried over Na_2SO_4 and filtered. The residue was obtained after rotatory evaporation to remove ethyl acetate and it was further purified by RP-HPLC (column: CAPCELL PAK C8 (150 mm × 20 mm I.D.), eluent: acetonitrile/H2O (0.1% TFA) = 40:60 to 60:40 (linear gradient over 40 min), flow 5.0 mL/min. **MTpcFFF** was obtained as a white powder. ^1H NMR (600 MHz, CD_3CN) δ = 7.30 – 7.19 (m, 18H), 7.05 (s, 1H), 6.90 (d, J=8.0 Hz, 2H), 6.28 (s, 1H), 4.60 – 4.51 (m, 2H), 4.32 – 4.24 (m, 1H), 3.14 – 3.07 (m, 2H), 3.06 – 3.02 (m, 1H), 2.98 – 2.90 (m, 1H), 2.90 – 2.84 (m, 1H), 2.80 – 2.74 (m, 1H), 2.43 (s, 3H). ^{13}C NMR (151 MHz, CD_3CN) δ = 172.9, 171.7, 155.4, 149.6, 138.2, 136.2, 130.3, 129.3, 128.3, 127.6, 123.2, 57.6, 55.3, 54.8, 38.4, 37.8, 30.3, 16.2. LCMS (ESI) calcd for $C_{35}H_{35}N_3O_6S$ $[M+H]^+$: 626.23, found 626.17.

^{1}H NMR study of oxidation and hydrolysis of 1 with and without *Ci*VCPO

In a NMR tube, 2.90 mg of **1** was dissolved in citrate buffer (CB, 50 mM, pH=6.2, 165 mM NaCl)/D_2O 9:1. Sodium trimethylsilylpropanesulfonate (DSS) was added in stoichiometric amount to **1** and used as NMR standard. To this solution, 0.0, 0.7 or 7.7 µL of the 65.0 µM *Ci*VCPO stock solution in Tris/H_2SO_4 buffer (50 mM, pH 8.2) was added to reach respectively of 0.0, 0.1 and 1.0 µM of *Ci*VCPO. The total volume of each experiment was 500 µL. Then, 15.0 µL of 3.0 wt% H_2O_2 was added to the tube and the reaction was followed by ^{1}H NMR in PRESAT mode over time. All the experiments were performed in duplicates to obtain mean and standard deviation values.

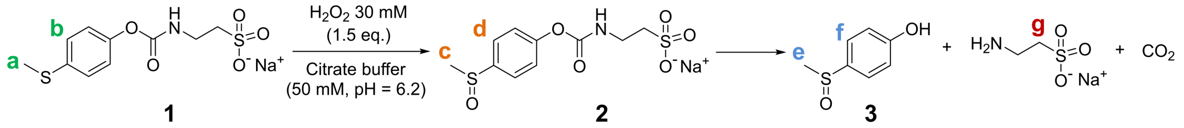

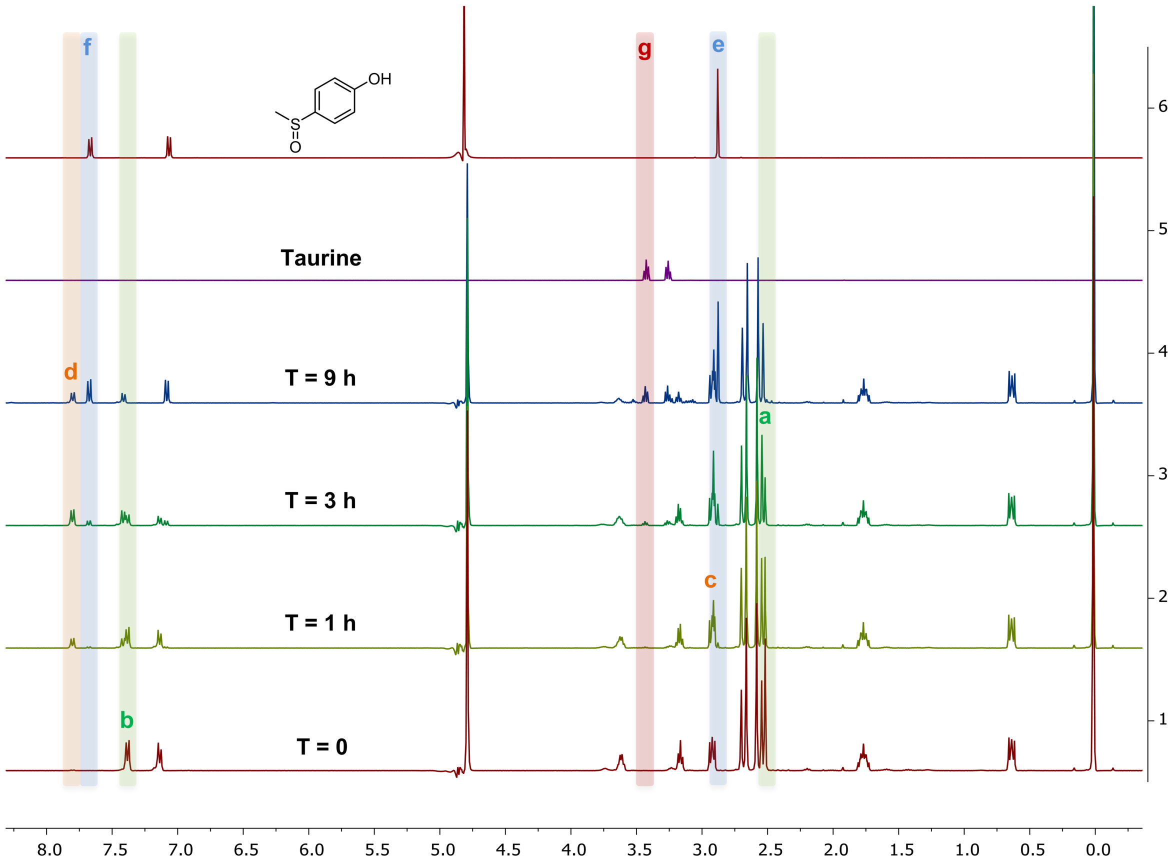

Figure S1. ^{1}H NMR spectra over time at 37 °C of **1** in CB/D_2O 9:1 upon the addition of 30 mM H_2O_2

^{1}H NMR study of oxidation and hydrolysis of 1 with and without r*Aae*UPO

In a NMR tube, 2.90 mg of **1** was dissolved in phosphate buffer (PB, 50 mM, pH=7.0)/D_2O 9:1. Sodium trimethylsilylpropanesulfonate (DSS) was added in stoichiometric amount to **1** and used as NMR standard. To this solution, 0.0, 0.6 or 6.0 µL of the 83.9 µM r*Aae*UPO stock solution in potassium phosphate buffer (20 mM, pH 7.0) was added to reach respectively of 0.0, 0.1 and 1.0 µM of r*Aae*UPO. The total volume of each experiment was 500 µL Then, 15.0 µL of 3.0 wt% H_2O_2 was added to the tube and the reaction at 37 °C was followed by ^{1}H NMR in PRESAT mode over time. All the experiments were performed in duplicates to obtain mean and standard deviation values.

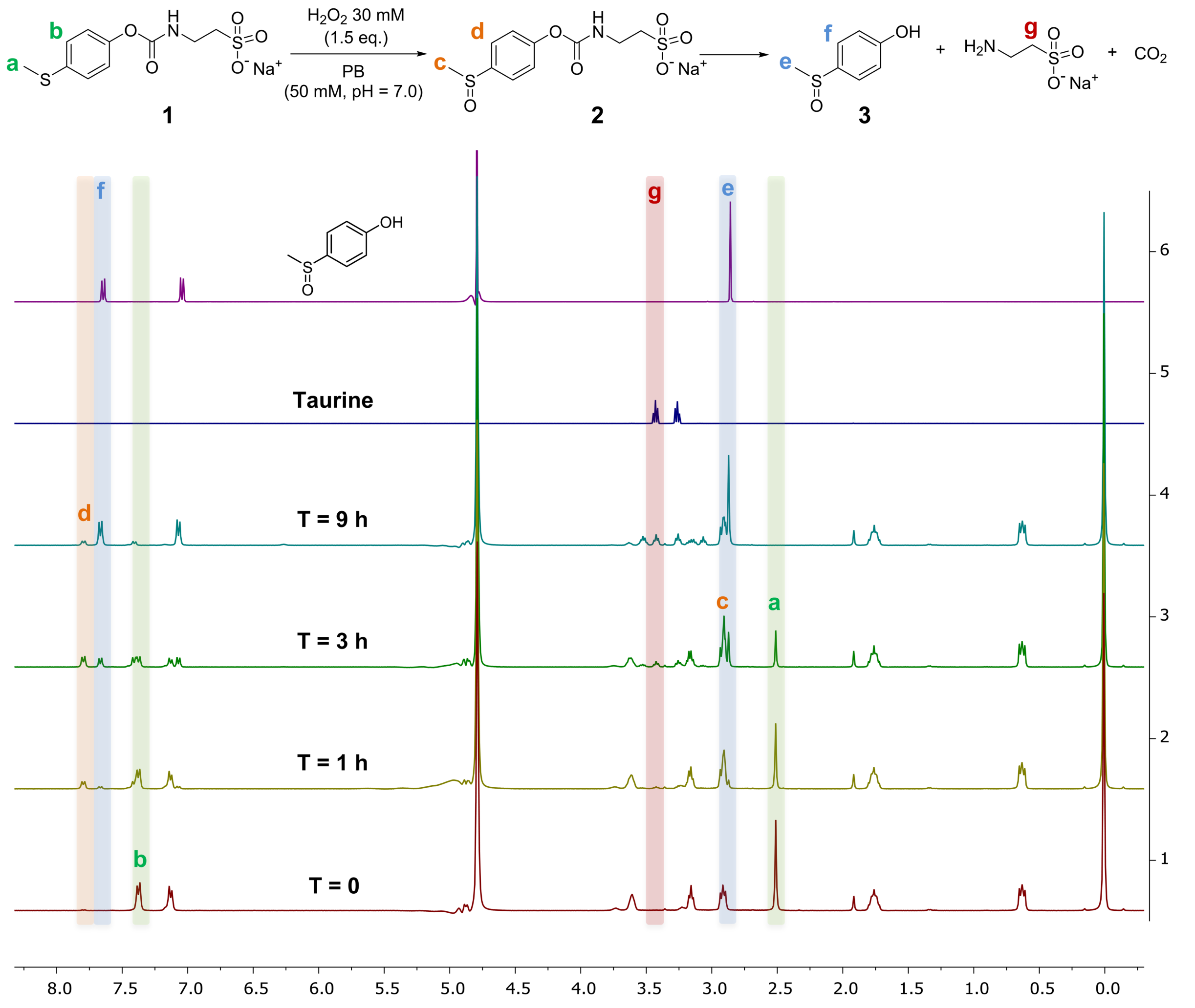

Figure S2. ^{1}H NMR spectra over time at 37 °C of **1** in PB/D_2O 9:1 upon the addition of 30 mM H_2O_2.

Optical microscopy of MTpcFF hydrogels

MTpcFF hydrogels were prepared dissolving 1.0 mg **MTpcFF** in 5.0 µL of DMSO, then adding 95.0 µL of phosphate buffer (PB, 50 mM, pH=7.0). **MTpcFF** gels in citrate buffer (CB, 50 mM, pH=6.2) were prepared dissolving 0.75 mg **MTpcFF** in 5.0 µL of DMSO, then adding 95.0 µL of the buffer on top. After formation of stable gels, a portion of **MTpcFF** hydrogels was placed in the center of a glass slide, then a glass cover slip was positioned on top of the sample and observed in the microscope. Optical images were processed using ImageJ.

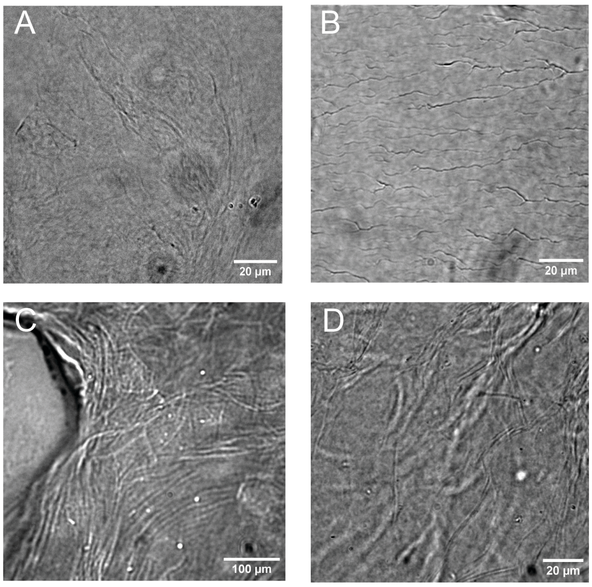

Figure S3. Optical images of **MTpcFF** hydrogels. Optical images of **MTpcFF** (0.75 wt%) hydrogel in CB (Scale bar = 20 µm) at 63X magnification show fibers of about 1.0 µm diameter, which A) intersect when surrounded by water and B) assume a parallel orientation after the evaporation of water from the sample. **MTpcFF** (1.0 wt%) hydrogel in PB at C) 20X magnification (Scale bar = 100 µm) and D) 63X magnification (Scale bar = 20 µm) present bundles of fibers with diameter up to 3.0 µm.

Cryo-EM of MTpcFF hydrogels

MTpcFF hydrogels were prepared dissolving 1.0 mg **MTpcFF** in 5.0 µL of DMSO, then adding 95.0 µL of phosphate buffer (PB, 50 mM, pH=7.0). **MTpcFF** gels in citrate buffer (CB, 50 mM, pH=6.2) were prepared dissolving 0.75 mg **MTpcFF** in 5.0 µL of DMSO, then adding 95.0 µL of the buffer on top. Cryo-TEM images were obtained by placing 4.0 µL of the sample onto Quantifoil 300 mesh Cu R1.2/1.3 grids. The drop was blotted to obtain a thin layer on the grid, and vitrified by rapid immersion in liquid ethane (Leica EM GP version 16222032) with a Vitrobot plunger. The grid was finally inserted into a cryo-holder (Gatan model 626) and then transferred to the Jeol JEM 1400 TEM. Cryo-EM images were processed using ImageJ.

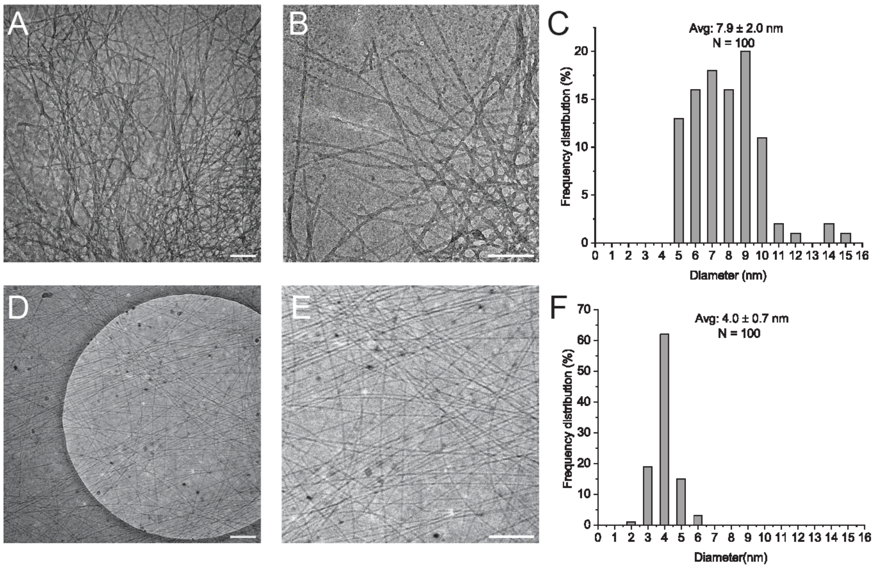

Figure S4. A, B) Cryo-EM images of **MTpcFF** (0.75 wt%) hydrogel in CB (scale bar = 200 nm) show dense fibrous network. C) Frequency distribution based on Cryo-EM images analysis of **MTpcFF** (0.75 wt%) hydrogel in CB results in an average fiber diameter of 7.9 ± 2.0 nm. D,E) Cryo-EM images of **MTpcFF** (1.0 wt%) hydrogel in PB (scale bar = 200 nm) show ordered fibers. F) Frequency distribution based on Cryo-EM images analysis of **MTpcFF** (1.0 wt%) hydrogel in PB results in an average fiber diameter of 4.0 ± 0.7 nm.

MTpcFF hydrogels preparation with and without *Ci*VCPO (tube inversion)

In a 1.5 mL screw cap vial, **MTpcFF** was dissolved in 5.0 μL of DMSO. Then, 95.0 μL of citrate buffer (CB, 50 mM, pH=6.2) was slowly added. Each vial was stirred by vortexing for 3 seconds, capped, placed on a stable surface and left undisturbed overnight. The gelation was evaluated turning the vial upside down. Mixtures of **MTpcFF** formulations that were not able to form a gel and stayed liquid and clear were classified as “sol”. Hydrogels which partially collapsed upon turning the vial upside down were considered “weak gels”. Hydrogels that stayed intact upon turning the vial upside down, but appeared clear, were considered “clear gels”. Hydrogels that were intact after turning the vial upside down, but appeared more opaque than the “clear gels” were classified as “opaque gels” (see Figure S5). The gelation experiments were performed in duplicates.

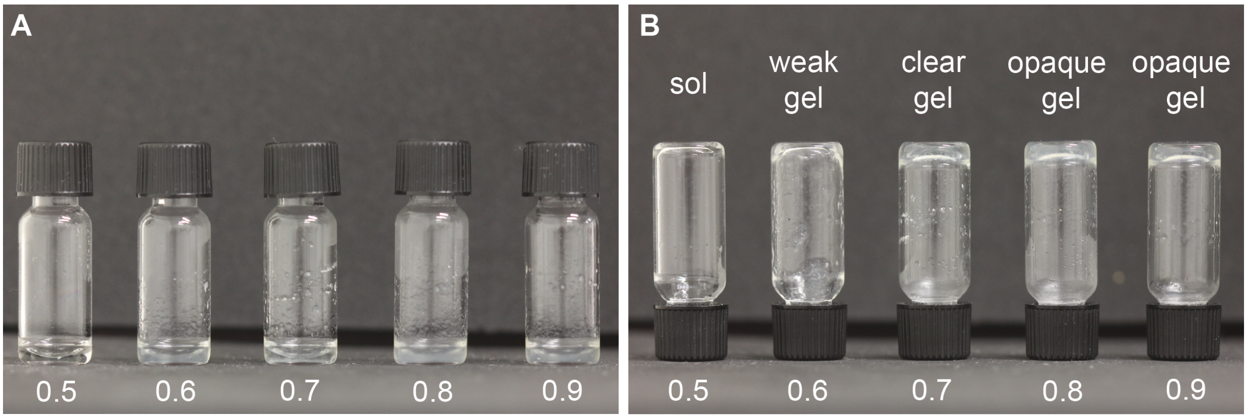

Figure S5. Tube-inversion tests of **MTpcFF** in citrate buffer at pH = 6.2. From left to right 0.5, 0.6, 0.7, 0.8, 0.9 wt% **MTpcFF** right after stirring (A) and after overnight gelation (B).

MTpcFF hydrogels preparation with and without r*Aae*UPO (tube inversion)

In a 1.5 mL screwed vial, **MTpcFF** was dissolved in 5.0 µL of DMSO. Then, 95.0 µL of phosphate buffer (PB, 50 mM, pH=7.0) was slowly added. Each vial was stirred by vortexing for 3 seconds, capped, placed on a stable surface and left undisturbed overnight. The gelation was evaluated turning the vial upside down. Mixtures of **MTpcFF** formulations that were not able to form a gel and stayed liquid and clear were classified as "sol". Hydrogels which partially collapsed upon turning the vial upside down were considered "weak gels". Hydrogels that stayed intact upon turning the vial upside down, but appeared clear, were considered "clear gels". Hydrogels that were intact after turning the vial upside down, but appeared more opaque than the "clear gels" were classified as "opaque gels" (see Figure S6). The gelation experiments were performed in duplicates. The gelation experiments were performed in duplicates.

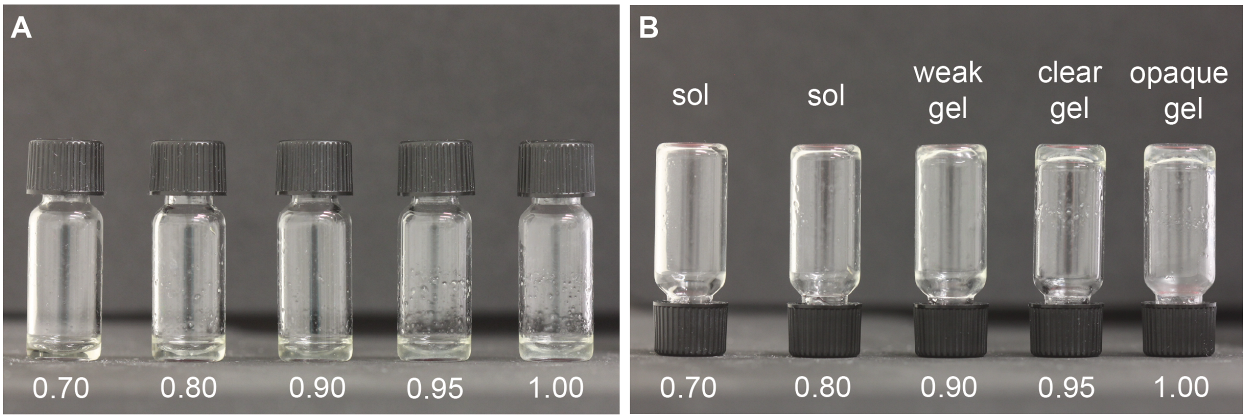

Figure S6. Tube-inversion tests of **MTpcFF** in phosphate buffer at pH = 7.0. From left to right 0.7, 0.8, 0.9, 0.95, 1.0 wt% **MTpcFF** right after stirring (A) and after overnight gelation (B)

Stability of MTpcFF hydrogels in solution (tube inversion)

To the **MTpcFF** gels prepared as described above, 500 or 200 µL of the corresponding buffer was added on top. The vials were left standing over time and the gel stability was evaluated turning the vials upside down at different times.

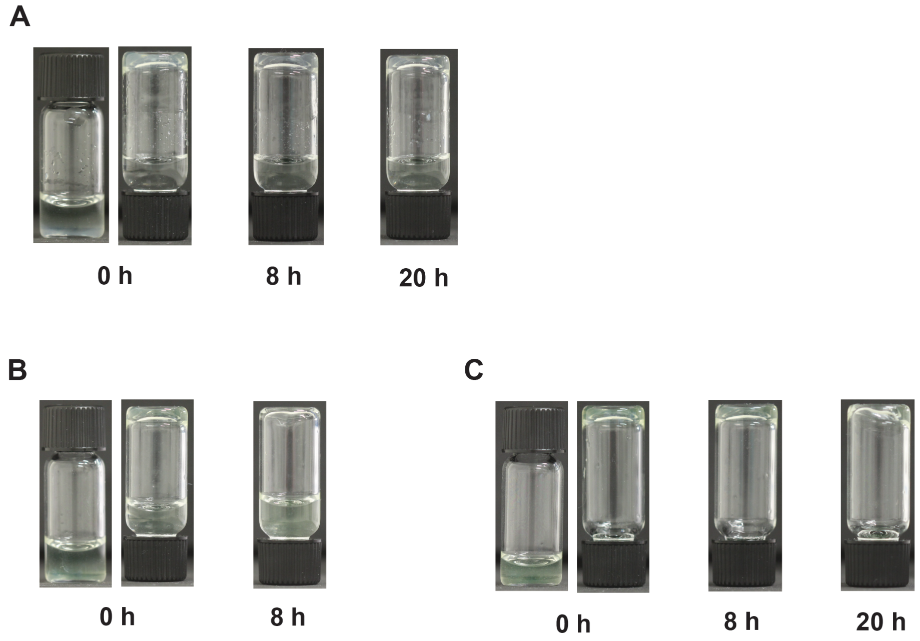

Figure S7. Photographs of stability tests of **MTpcFF** in solution. A) **MTpcFF** hydrogel (0.75 wt%) prepared in citrate buffer at pH = 6.2 after the addition of 500 µL of citrate buffer. The hydrogel was stable for 20 h in solution. B) **MTpcFF** hydrogel (1.0 wt%) prepared in phosphate buffer at pH = 7.0 after the addition of 500 µL of phosphate buffer. After 8 h only a thin layer of gel is left at the bottom of the vial. C) **MTpcFF** hydrogel (1.0 wt%) prepared in phosphate buffer at pH = 7.0 after the addition of 200 µL of phosphate buffer. The gel remained stable for 8 h and partially collapsed at 20 h.

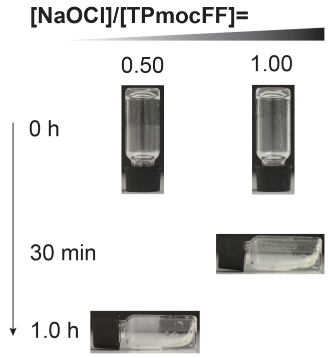

Figure S8. Photographs of **MTpcFF** hydrogels (0.75 wt%) in CB (50 mM, pH = 6.2) after the addition of 1.0 and 0.5 eq. NaOCl. The results are in line with those obtained after the addition of 140 mM NaCl and either 1.0 or 0.5 eq. H_2O_2 in presence of 1.0 µM *Ci*VCPO.

HPLC analysis of MTpcFF hydrogels upon addition of H_2O_2

To the solution or partially destructed **MTpcFF** hydrogel obtained as explained above was added 900 µL of acetonitrile 5 h after the addition of 1.0, 0.5 and 0.25 equivalents of H_2O_2 and 8 h after the addition of 0.1 and 0.0 equivalents of H_2O_2. This solution was diluted two times and an aliquot (10.0 µL) was analysed by RP-HPLC (Column: Lichrospher RP18-5 (150 × 4.6 mm, 5.0 µm). Eluent: A:B = 20:80 to 80:20 (A: Acetonitrile/0.1%TFA, B: H2O/0.1%TFA), linear gradient over 40 min, flow rate = 0.5 mL min–1) at a detection wavelength of 220 nm.

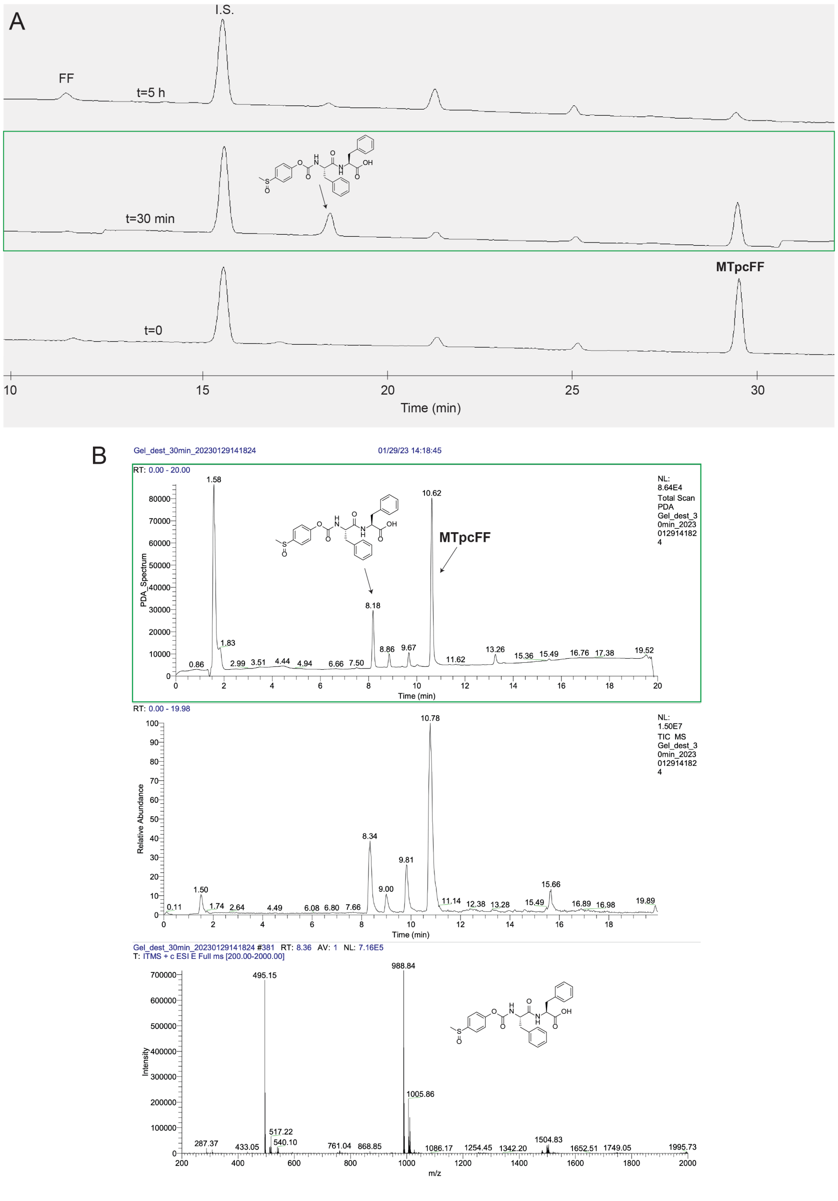

Figure S9. A) HPLC analysis of **MTpcFF** hydrogel before oxidation (bottom) and 30 min (middle) and 5 h (top) after the addition of H_2O_2 ($[H_2O_2]$/[**MTpcFF**] = 1.0) in presence of 1.0 µM *Ci*VCPO in CB. (Internal standard (I.S.): N-Methyl-p-toluenesulfonamide). B) LC-MS analysis of **MTpcFF** hydrogel 30 min after the addition of H_2O_2 ($[H_2O_2]$/[**MTpcFF**] = 1.0) in presence of 1.0 µM *Ci*VCPO in water.

Supplementary references

1. M. Ikeda, T. Tanida, T. Yoshii, K. Kurotani, S. Onogi, K. Urayama and I. Hamachi, *Nat. Chem.*, 2014, **6**, 511-518.
2. S. Dadhwal, J. M. Fairhall, S. K. Goswami, S. Hook and A. B. Gamble, *Chem. Asian J.*, 2019, **14**, 1143-1150.

Spectra of synthesized compounds

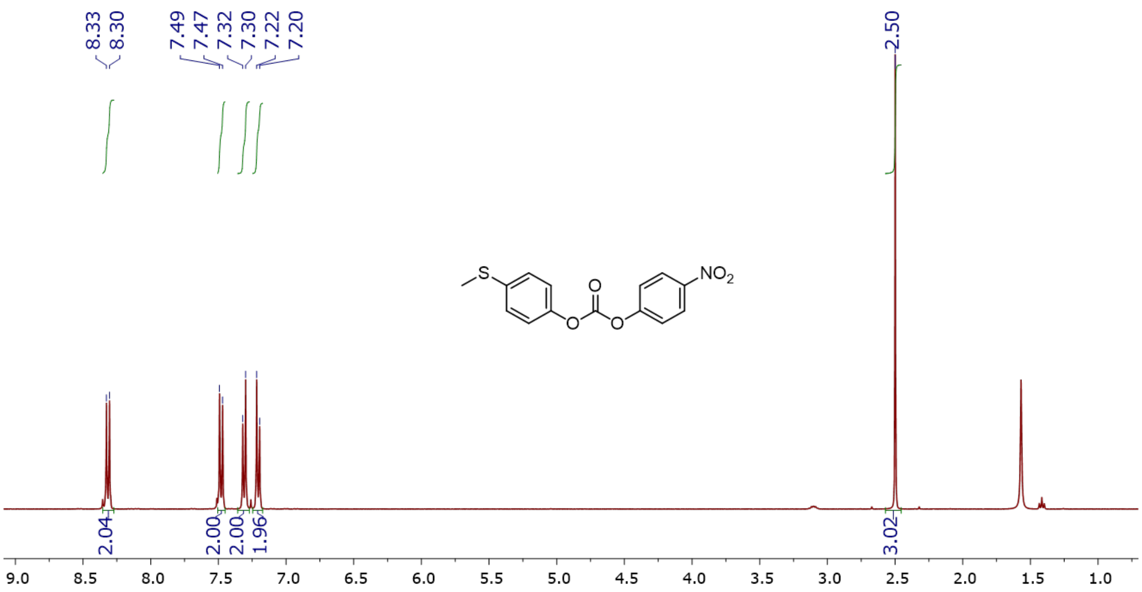

^{1}H NMR spectrum (400 MHz) of 4-(methylthio)phenyl 4-nitrophenyl carbonate in $CDCl_3$.

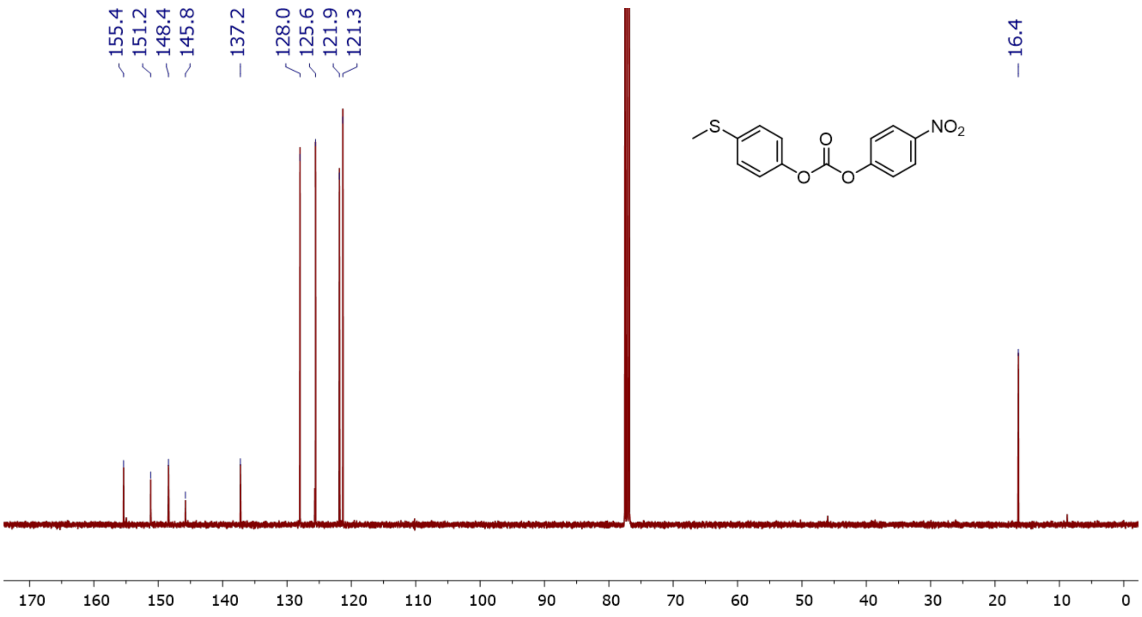

^{13}C NMR spectrum (101 MHz) of 4-(methylthio)phenyl 4-nitrophenyl carbonate in $CDCl_3$.

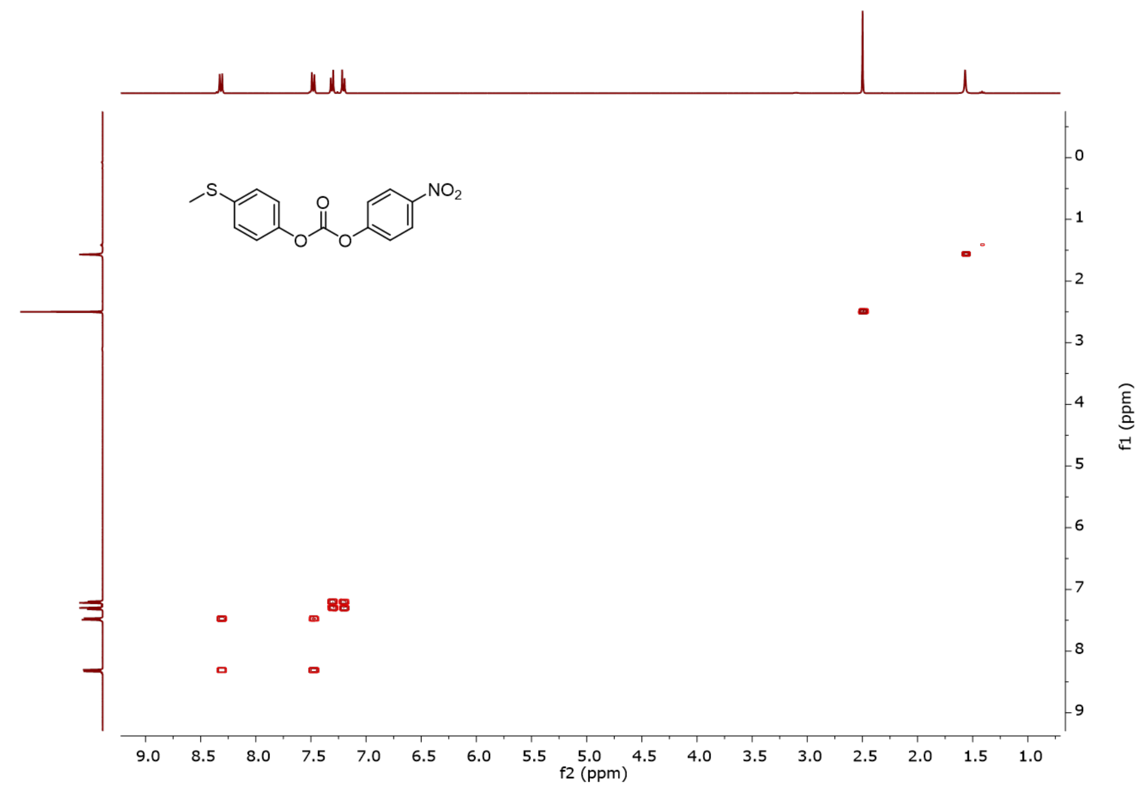

^{1}H-^{1}H-COSY spectrum of 4-(methylthio)phenyl 4-nitrophenyl carbonate in $CDCl_3$.

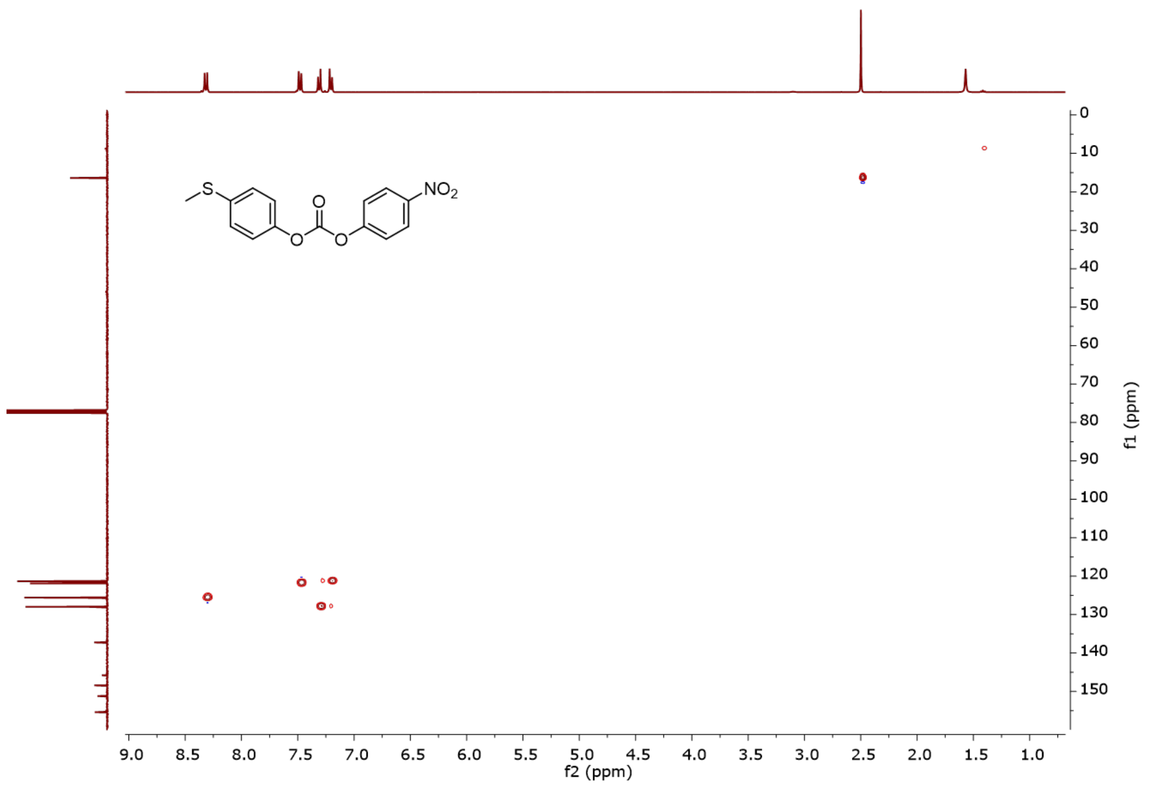

HSQC spectrum of 4-(methylthio)phenyl 4-nitrophenyl carbonate in $CDCl_3$.

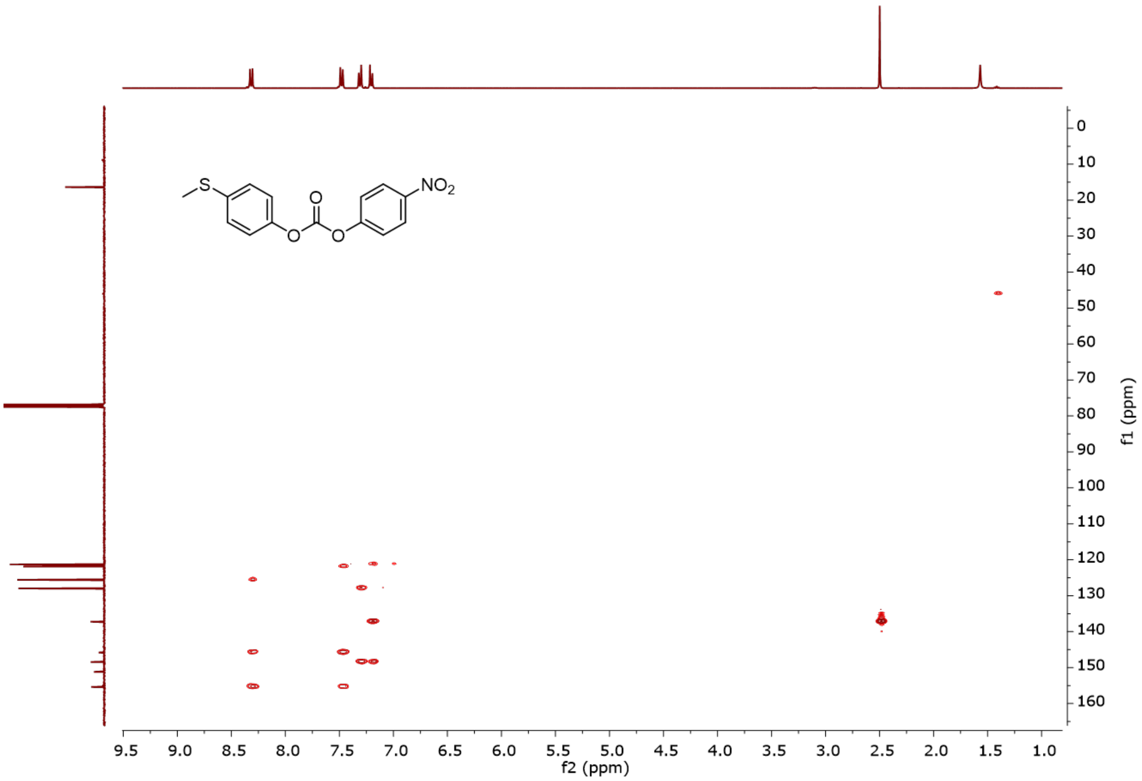

HMBC spectrum of 4-(methylthio)phenyl 4-nitrophenyl carbonate in $CDCl_3$.

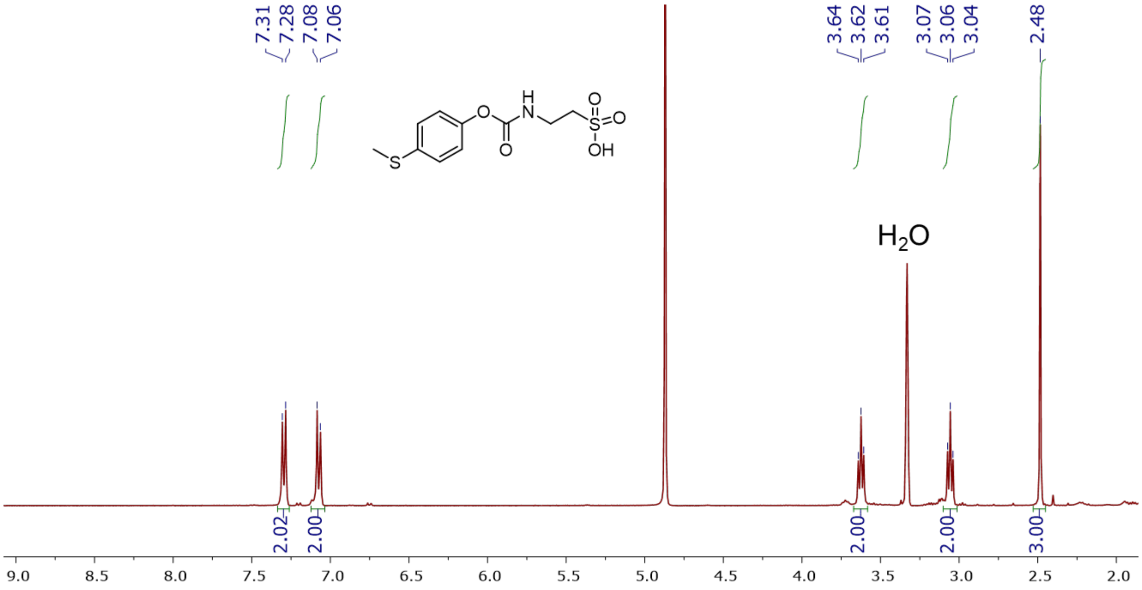

^{1}H NMR spectrum (400 MHz) of compound **1** in CD_3OD.

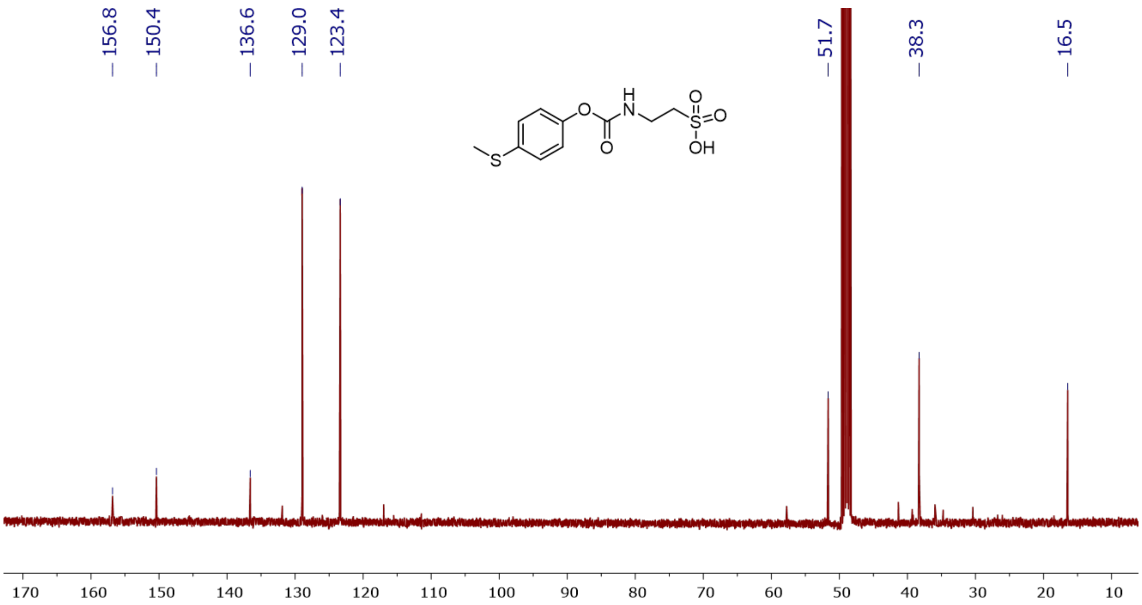

^{13}C NMR spectrum (101 MHz) of compound **1** in CD_3OD.

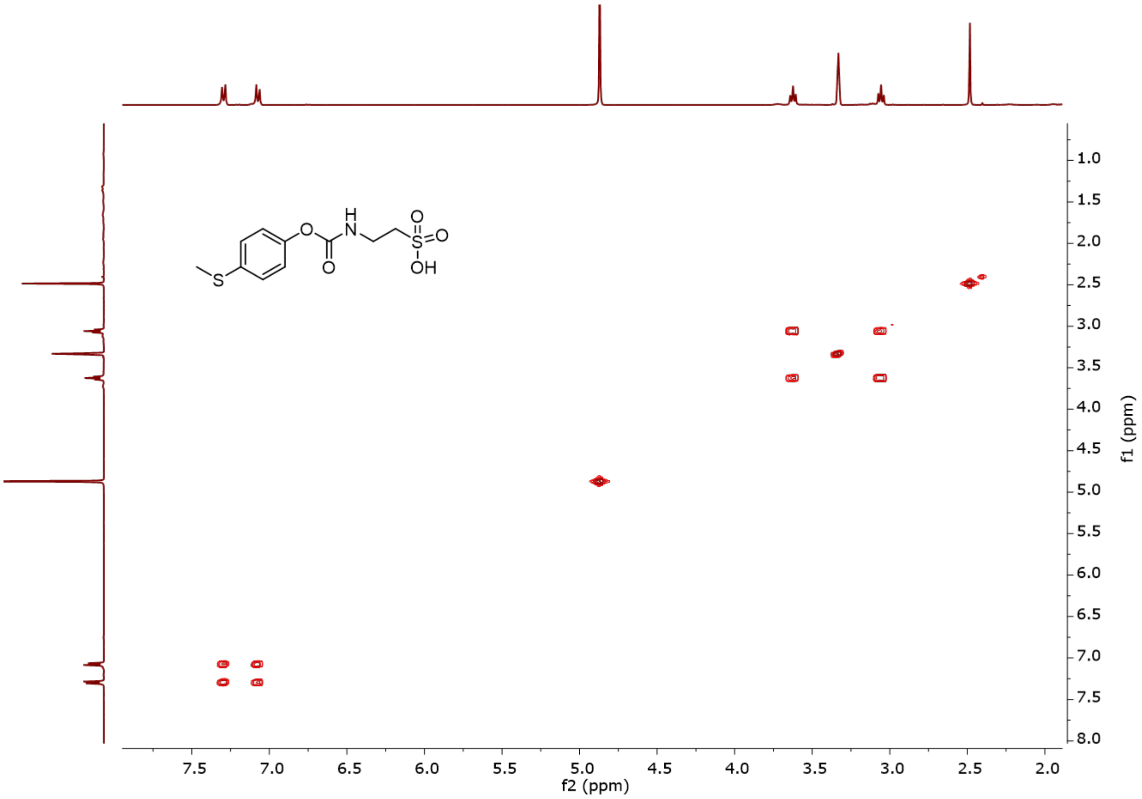

^1H-^1H-COSY spectrum of compound **1** in CD_3OD.

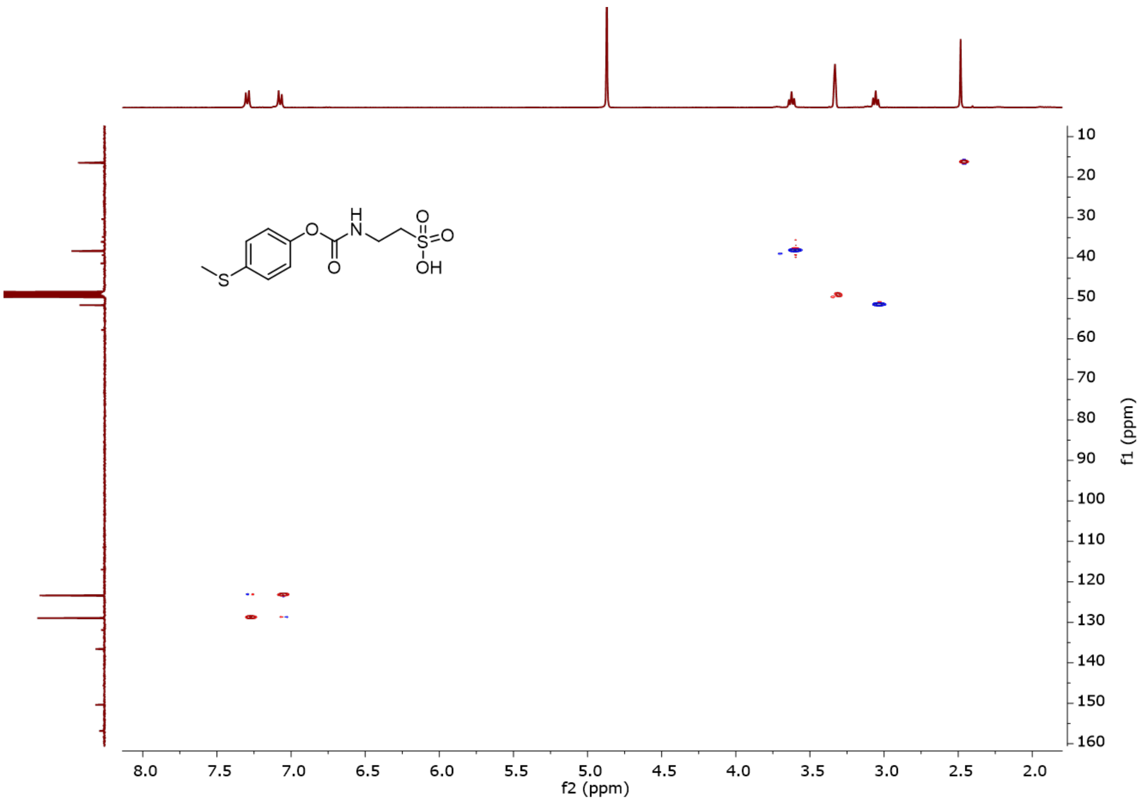

HSQC spectrum of compound **1** in CD_3OD.

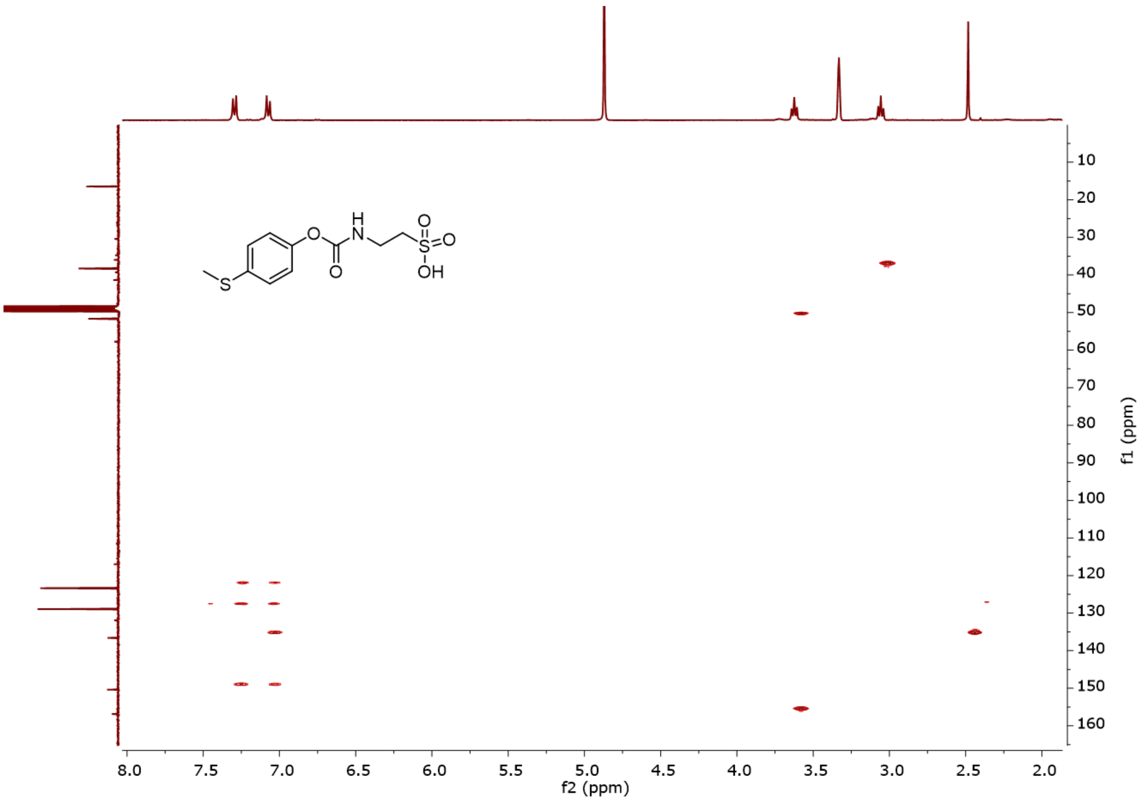

HMBC spectrum of compound **1** in CD_3OD.

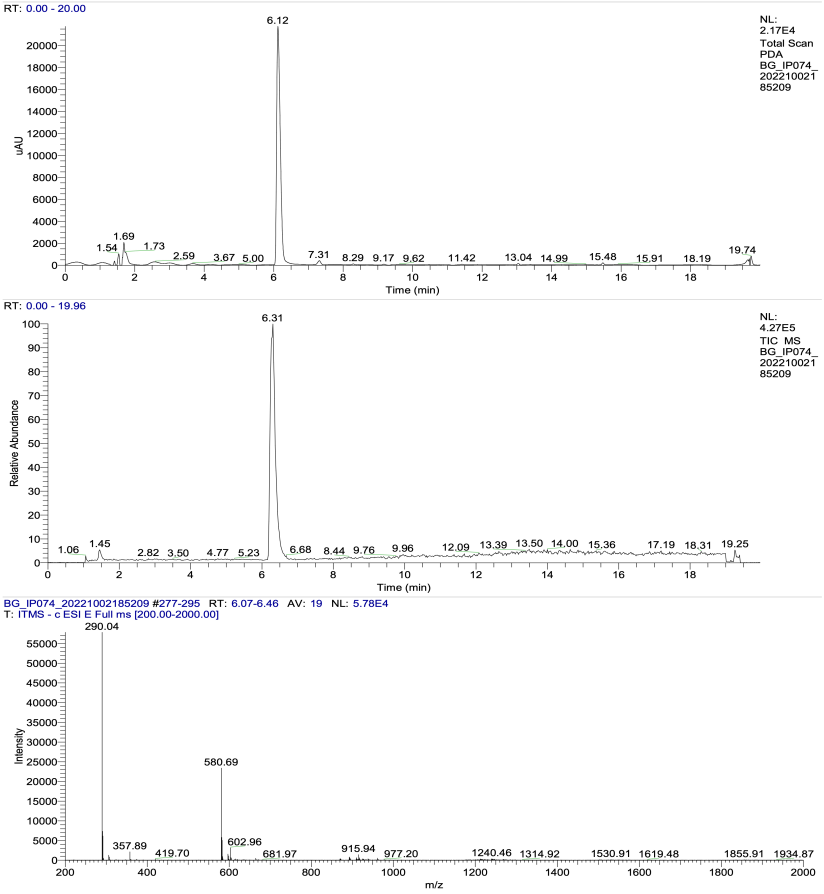

LC MS of compound **1**

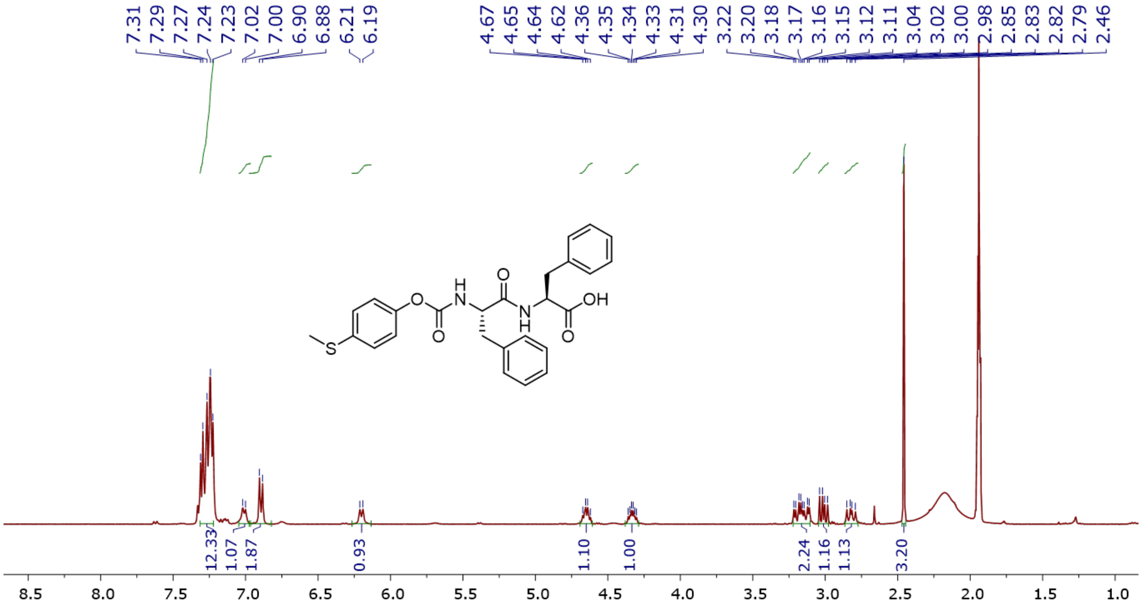

^{1}H NMR spectrum (400 MHz) of **MTpcFF** in CD_3CN.

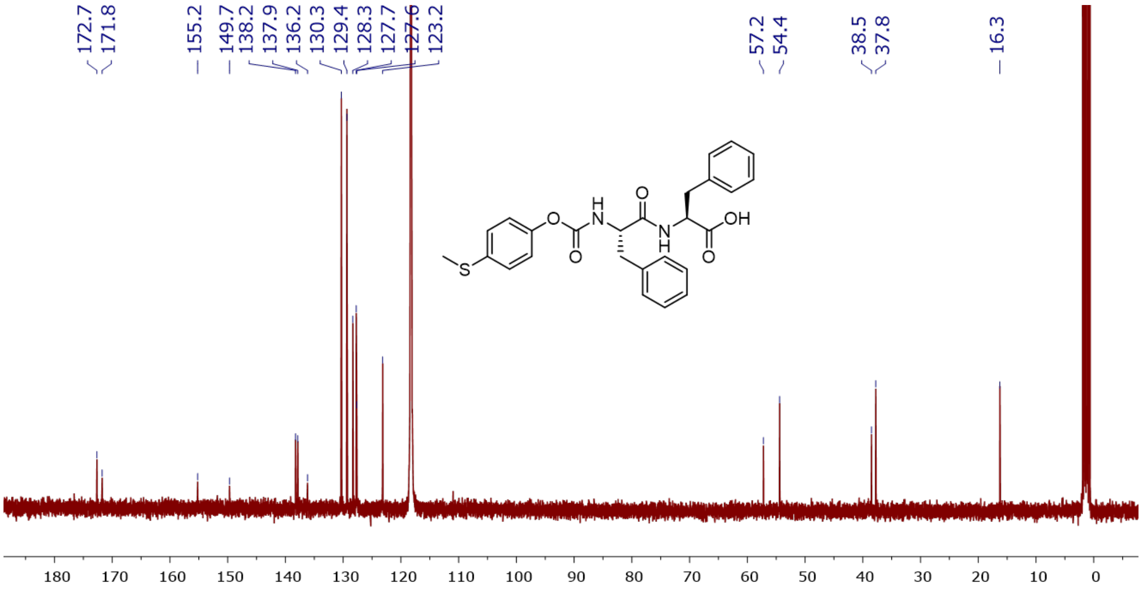

^{13}C NMR spectrum (101 MHz) of **MTpcFF** in CD_3CN.

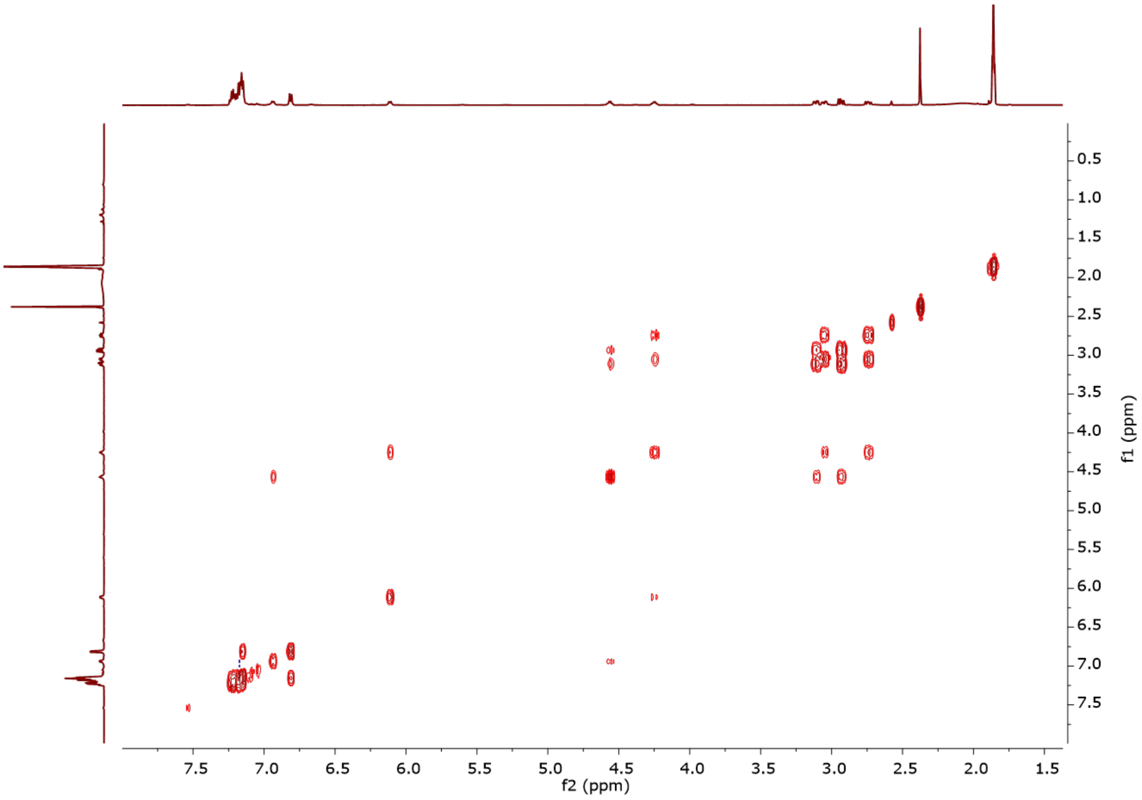

^1H-^1H-COSY spectrum of **MTpcFF** in CD_3CN.

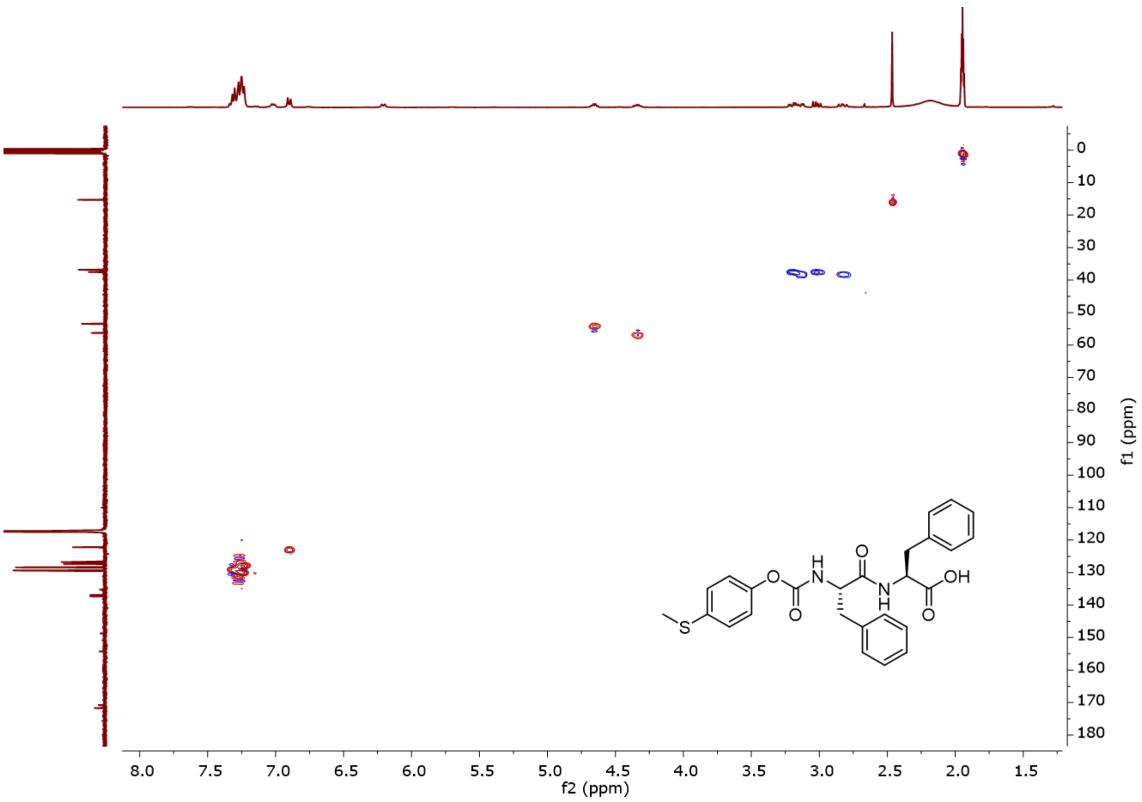

HSQC spectrum of **MTpcFF** in CD_3CN.

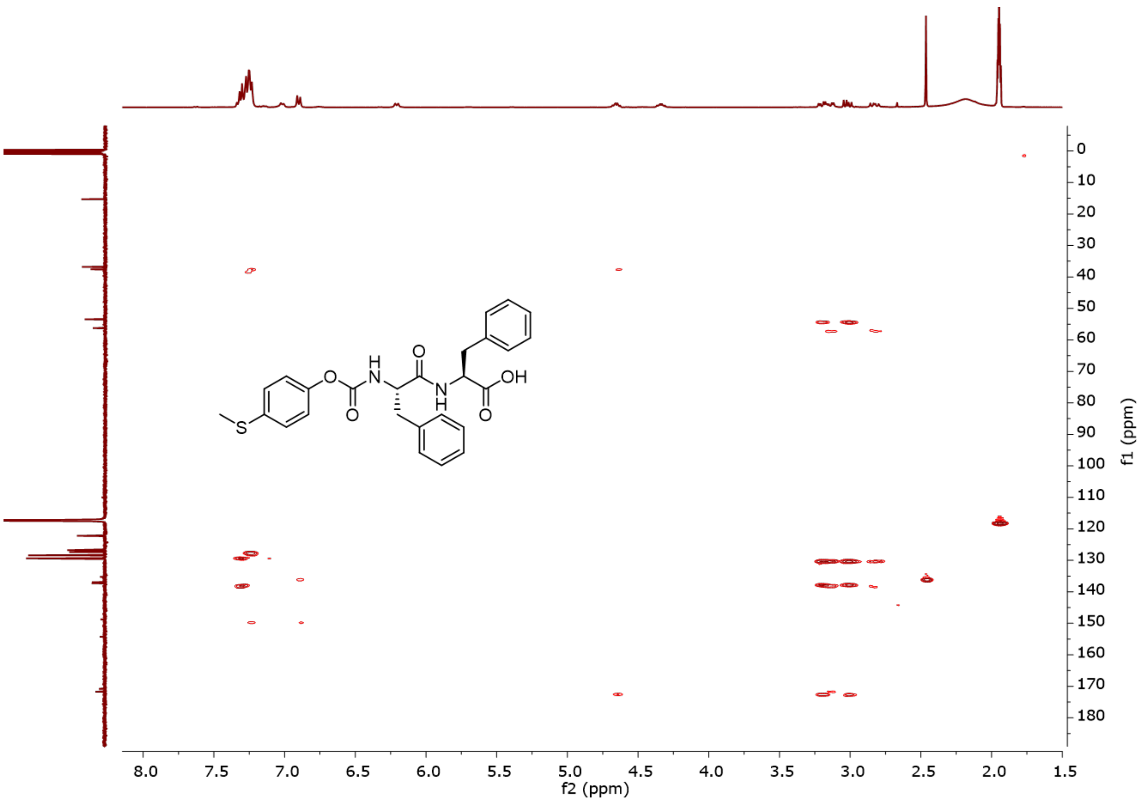

HMBC spectrum of **MTpcFF** in CD_3CN.

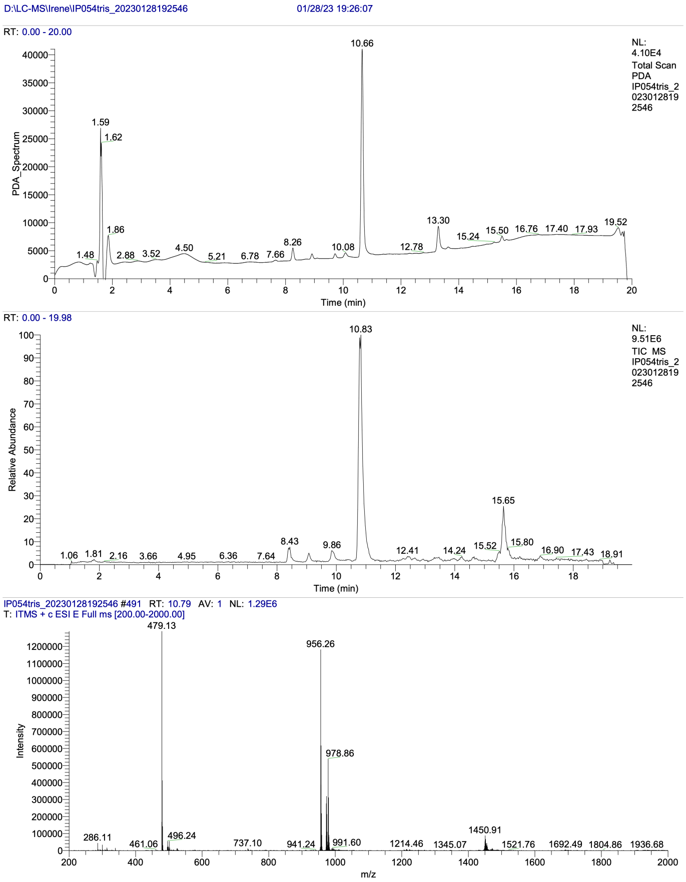

LC-MS spectra of **MTpcFF** in acetonitrile

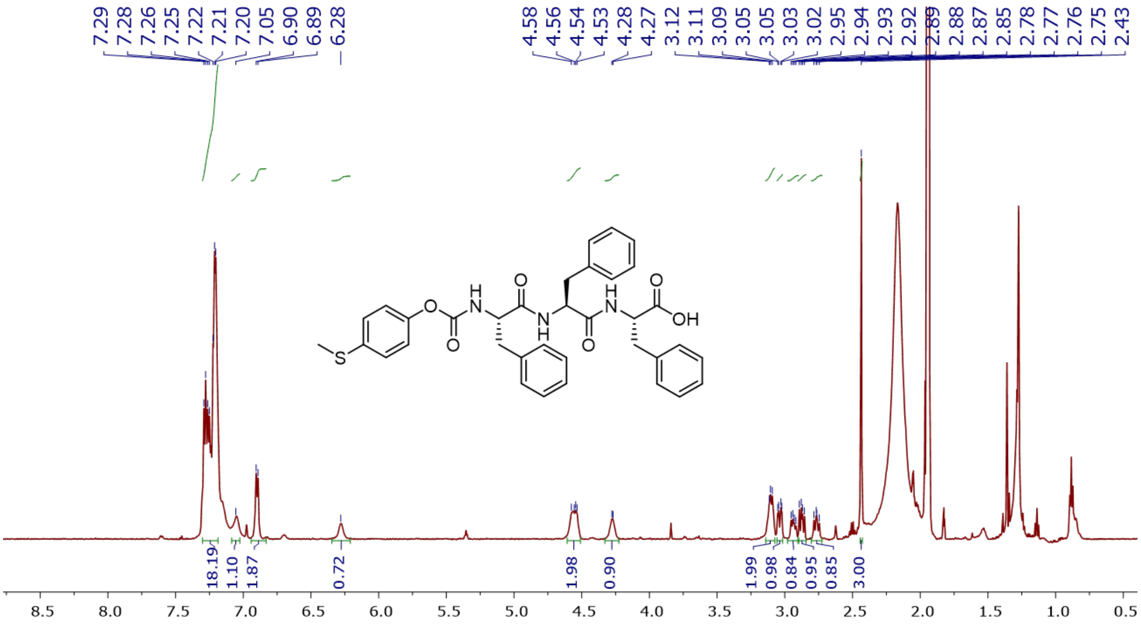

^{1}H NMR spectrum (600 MHz) of **MTpc-FFF** in CD_3CN.

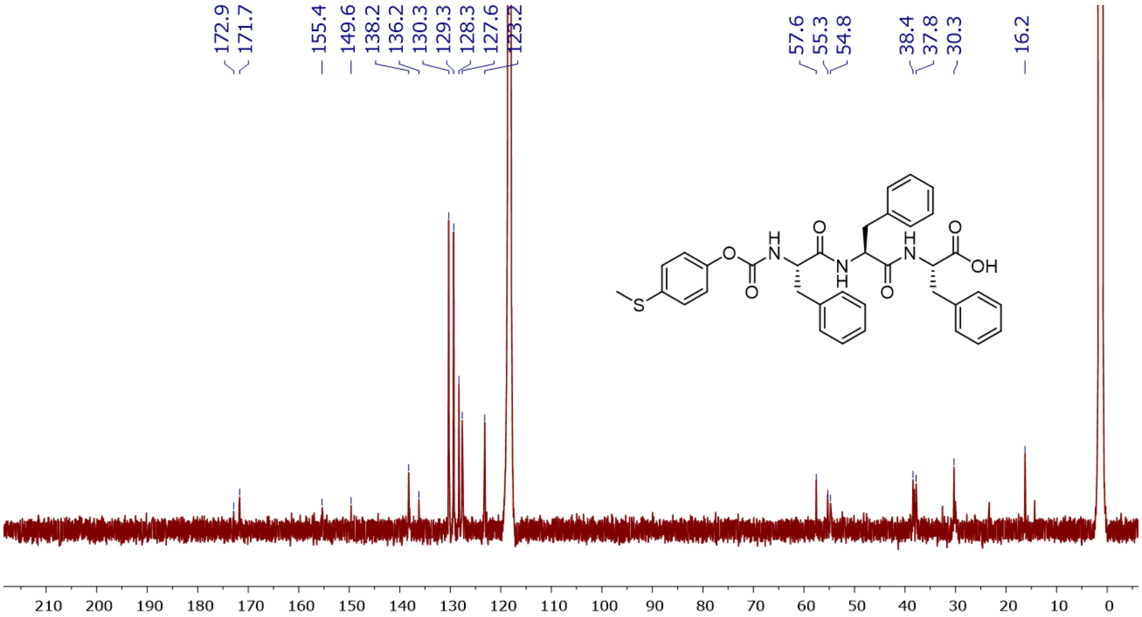

^{13}C NMR spectrum (151 MHz) of **MTpcFFF** in CD_3CN.

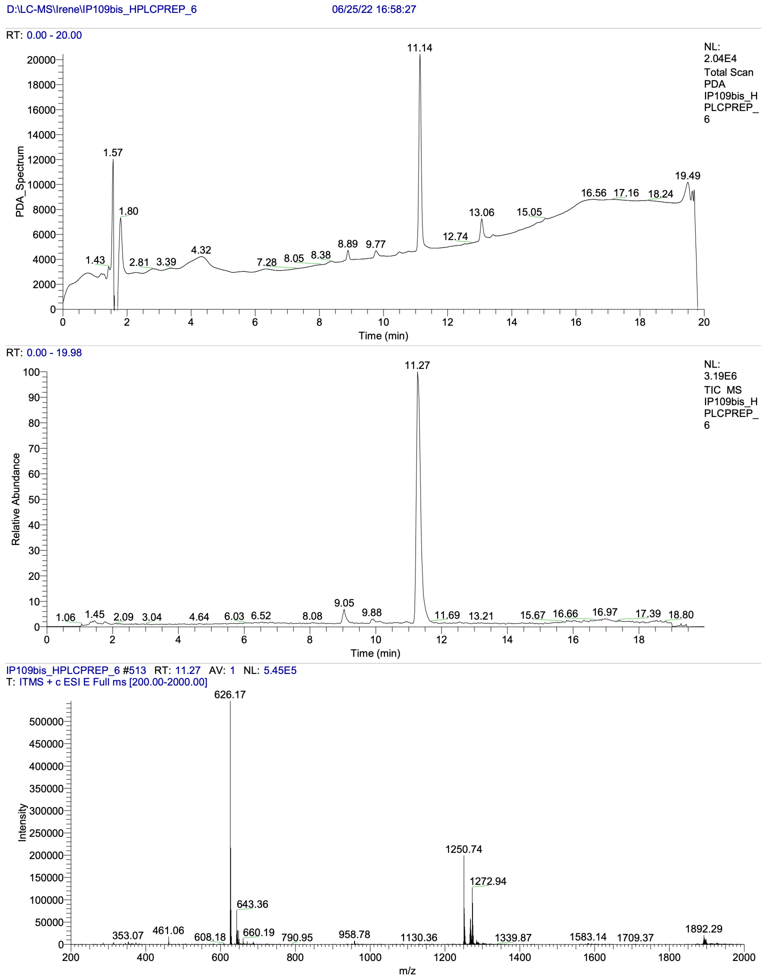

LC-MS spectra of **MTpcFFF** in acetonitrile
